# MYPT1 reduction is a pathogenic factor of erectile dysfunction

**DOI:** 10.1038/s42003-022-03716-y

**Published:** 2022-07-25

**Authors:** Wei Zhao, Jie Sun, Liang-Yu Yao, Dong Hang, Ye-Qiong Li, Cai-Ping Chen, Yu-Wei Zhou, Xin Chen, Tao Tao, Li-Sha Wei, Yan-Yan Zheng, Xie Ge, Chao-Jun Li, Zhong-Cheng Xin, Yang Pan, Xin-Zhu Wang, Wei-Qi He, Xue-Na Zhang, Bing Yao, Min-Sheng Zhu

**Affiliations:** 1 Jinling Hospital Department of Reproductive Medical Center affiliated Sch Med, State Key Laboratory of Pharmaceutical Biotechnology and Jiangsu Key Laboratory of Molecular Medicine, Medical School of Nanjing University, Nanjing, China; 2 Department of Urology, the First Affiliated Hospital of Nanjing Medical University, Nanjing, China; 3 Department of Epidemiology and Biostatistics, School of Public Health, Nanjing Medical University, Nanjing, China; 4 State Key Laboratory of Natural Medicines, China Pharmaceutical University, Nanjing, China; 5 Andrology Center, Peking University First Hospital, Peking University, Beijing, China; 6 School of Pharmacy, Nanjing University of Chinese Medicine, Nanjing, China; 7 Cambridge-Suda (CAM-SU) Genomic Resource Center, Soochow University, Suzhou, China

**Keywords:** Erectile dysfunction, Vasodilation

## Abstract

Erectile dysfunction (ED) is closely associated with smooth muscle dysfunction, but its underlying mechanisms remains incompletely understood. We here reported that the reduced expression of myosin phosphatase target subunit 1 (MYPT1), the main regulatory unit of myosin light chain phosphatase, was critical for the development of vasculogenic ED. Male MYPT1 knockout mice had reduced fertility and the penises displayed impaired erections as evidenced by reduced intracavernous pressure (ICP). The penile smooth muscles of the knockout mice displayed enhanced response to G-Protein Couple Receptor agonism and depolarization contractility and resistant relaxation. We further identified a natural compound lotusine that increased the MYPT1 expression by inhibiting SIAH1/2 E3 ligases-mediated protein degradation. This compound sufficiently restored the ICP and improved histological characters of the penile artery of *Mypt1* haploinsufficiency mice. In diabetic ED mice (db/db), the decreased expression of MYPT1 was measured, and ICP was improved by lotusine treatment. We conclude that the reduction of MYPT1 is the major pathogenic factor of vasculogenic ED. The restoration of MYPT1 by lotusine improved the function of injured penile smooth muscles, and could be a novel strategy for ED therapy.

## Abstract (web summary)

Reduced expression of myosin phosphatase target subunit 1 (MYPT1) is critical for the development of vasculogenic erectile dysfunction and a natural compound, lotusine, is found to increase MYPT1 expression and rescue penile smooth muscle function.

## Introduction

Erectile dysfunction (ED) is the inability to achieve or maintain a penile erection sufficient for satisfactory sexual intercourse^1^. Nearly 52% of men between 40-70 years of age are reported to have ED^2^, and the worldwide prevalence is predicted to reach 322 million in 2025^3^. An interesting clinical feature of ED is the close association with cardiovascular diseases (CVD)^4,5^, e.g. more than half of all cases of diabetes, hypertension, and atherosclerosis are complicated with ED^3–6^. Therefore, ED phenotype is usually regarded as the end result of various cardiovascular diseases, and many investigators even consider ED as a vascular disease.

Erection is accomplished via multiple processes^1,3^. In brief, the pudendal artery provides the penile body with a blood supply and branches to dorsal and central arteries penetrating the corpus cavernosal tissue^7,8^. Upon sexual stimulation, the penile nerve and endothelium release the nitric oxide (NO) necessary for relaxation of the penile muscles, resulting in the filling of the sinuses with blood and the restriction of venous outflow^3,7–9^. After intercourse, the penis returns to a flaccid state through the initiation of penile smooth muscle contraction by neural and local factors^9–11^. Therefore, the balance of contraction/relaxation of penile smooth muscle plays a key role in the process of erection. We thus guess the impairment of the smooth muscles may be a critical pathogenic event in ED genesis, however, the molecular targets in the process remained unclear.

Although the penile artery and corpus cavernosum smooth muscle (CCSM) are highly specific, with unique properties, these muscles share several features common to smooth muscles^11^. Smooth muscle contraction is mediated by multiple signals, including depolarization and G protein-coupled receptor (GPCR) agonists. Depolarization initiates contraction by L-type Cav_1.2_-regulated calcium influx, which activates myosin light chain kinase (MLCK) for regulatory myosin light chain (RLC) phosphorylation (RLCp)^12,13^. The resultant RLCp allows myosin to form cross-bridges to bind to actin filaments and results in force development^13–15^. GPCR agonists evoke contraction by sequential activation of Gαq/11 and phospholipase C (PLC). The resultant inositol 1,4,5-trisphosphate (IP3) mediates calcium release from the sarcoplasmic reticulum and hence induces MLCK activity. During this process, the PKC/CPI-17/myosin light chain phosphatase (MLCP) and Rho/ROCK/MLCP axes are also activated and mediate calcium-sensitized contraction^14–17^. The level of RLCp or relative activity of MLCK and MLCP determines the smooth muscle contractile status. MLCP is composed of three subunits: Myosin Phosphatase Target Subunit 1 (MYPT1), a PP1c catalytic core, and a 20 kD kinase with unknown function. MYPT1 regulates the activity of the MLCP holoenzyme primarily via physical interaction with the PP1c catalytic subunit^14,18^. Ablation of MYPT1 expression leads to altered contractile behaviors^16,19^, including hypercontractility and hyporelexation, whereas mutation of MYPT1 phosphorylation sites only slightly affects contraction^20^. This pattern implies that the protein level of intact MYPT1 rather than the phosphorylation status of MYPT1 is the primary factor regulating contractile behaviors. Here, we found that MYPT1 expression was reduced in the CC smooth muscle of ED patients. The ablation of MYPT1 altered the contractile properties of penile smooth muscle, resulting in ED genesis. MYPT1-deficient smooth muscle was more sensitive to GPCR agonists and more resistant to nitric oxide-mediated relaxation. Pharmacological intervention with Lotusine, a compound with potent activity toward upregulating MYPT1 expression, restored penile erectile function. Our results thus revealed an essential role of MYPT1 in ED genesis. We thus proposed that contractile dysfunction of vascular smooth muscle might be a key mechanism of vasculogenic ED and upregulation of MYPT1 expression served as an novel therapeutic or preventive strategy of ED.

## Results

### Decreased MYPT1 expression in penile smooth muscles is associated with ED

**Fig. 1:**
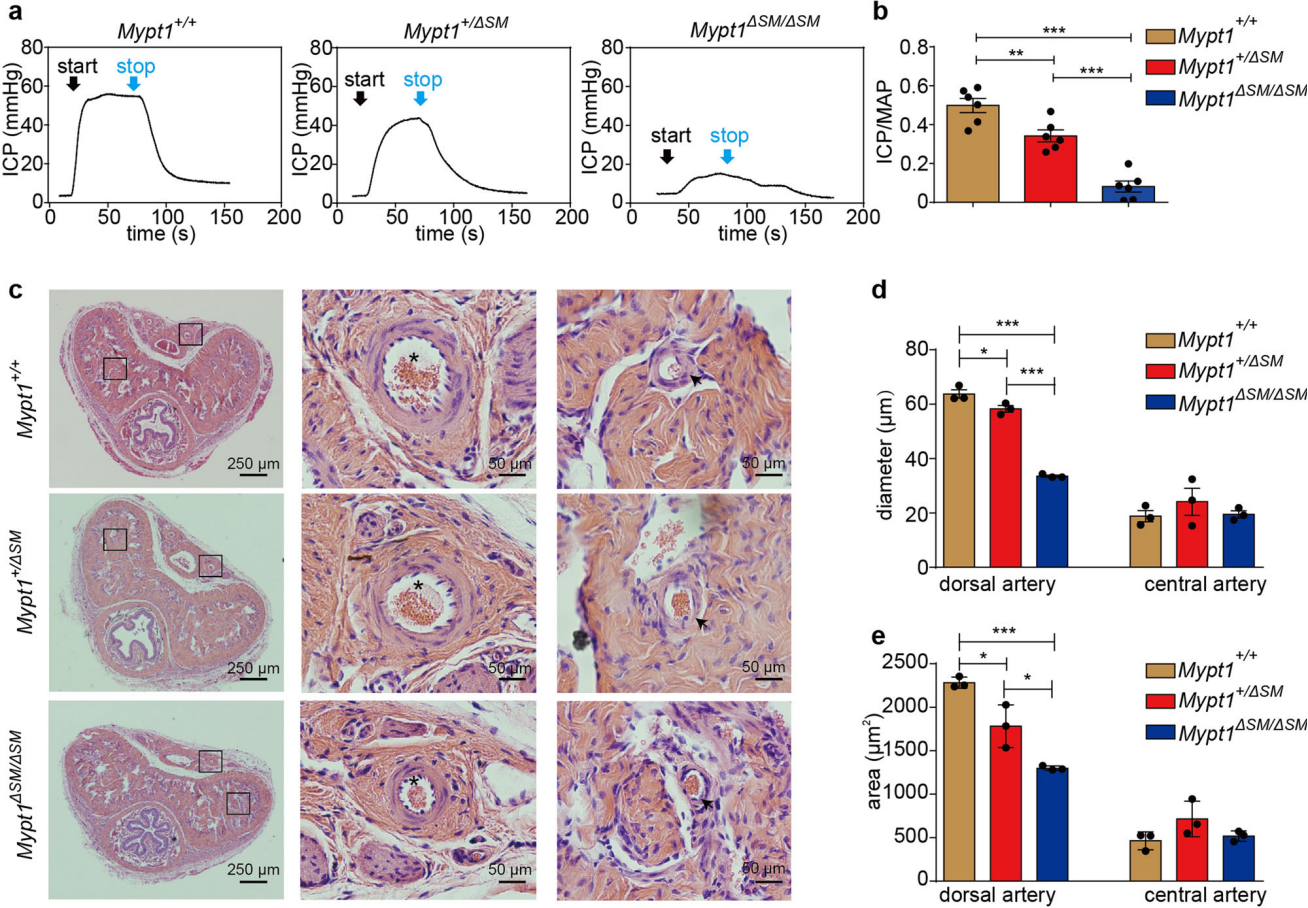
MYPT1-deficient penises showed a reduced ICP response to EFS and reduced arterial sizes. **a** Representative ICP tracings after stimulation of *Mypt1*^*+/+*^, *Mypt1*^*+/*Δ*SM*^, and *Mypt1*^Δ*SM /ΔSM*^ penises with 5 V/12 Hz for 1 min. **b** Quantification of the ICP/MAP values of the three groups (*n* = 6). **c**–**e**, H-E staining showed a decreased diameter and area of penile arteries. Quantitation of the diameter (**d**) and the area (**e**) of dorsal and central arteries (*n* = 3). “asterisk” represents dorsal artery; “arrow” represents helicine arteriole. The bars indicate the mean values ± SEMs; *p < 0.05, ***p < 0.001; *One-way ANOVA*.

To determine whether the level of MYPT1 protein is associated with penile erection, we first assessed the penile function in a mouse line with smooth muscle-specific deletion of MYPT1. The pup production of *Mypt1*^*+/+*^, *Mypt1*^*+/*Δ*SM*^ and *Mypt1*^Δ*SM/*Δ*SM*^ mice strikingly differed. All of the matings of 8 female with 8 male *Mypt1*^*+/+*^ mice produced 288 pups in 52 litters, and all of the matings of 8 female wild-type C57BL/6 mice with 8 males of *Mypt1*^*+/*Δ*SM*^ mice produced 246 pups in 44 litters. However, the matings of 6 female wild-type mice with 6 male *Mypt1*^Δ*SM/*Δ*SM*^ mice produced 10 pups in 2 litters (Table 1). These results show that male *Mypt1*^Δ*SM/*Δ*SM*^ mice presented subfertility, with small litter and pup numbers. To test whether subfertility was caused by an alteration in sexual desire, we evaluated sexual behaviors, including sniffing, mounting, grooming, and ejaculation within 45 min. The *Mypt1*^*+/+*^, *Mypt1*^*+/*Δ*SM*^ and *Mypt1*^Δ*SM/*Δ*SM*^ mice presented a comparable frequency of sexual behaviors (Table 2). No vaginal plug was observed in wild-type females that mated with *Mypt1*^Δ*SM/*Δ*SM*^ mice, implying no ejaculation of semen. This was unlikely caused by urethra impairment because the MYPT1-deficient mice had normal urination and intact urethra structure (Fig. 1). We then detected the sperm quality and the level of sexual hormone. The sperm concentration and sperm motility showed no difference among *Mypt1*^*+/+*^, *Mypt1*^*+/*Δ*SM*^ and *Mypt1*^Δ*SM/*Δ*SM*^ mice (Table 3), while the level of total testosterone and morphology of testis were also equal (Supplementary Fig. 1a-b). The morphology examination showed that the sperm cells from the *Mypt1*^Δ*SM/*Δ*SM*^ mice had typical structures of heads, tails and bodies, which were comparable to the control mice (Supplementary Fig. 2). Thus, the subfertility of *Mypt1*^Δ*SM/*Δ*SM*^ males might not be caused by an alteration in spermatozoa or neither sexual desire. Nevertheless, our present data could not completely rule out other possibilities beyond ED for the lower fertility, which was necessarily investigated in the future.

**Table 1:** Characterization of the birth patterns of different genotyping mice.

	*Mypt1*^*+/+*^	*Mypt1*^*+/*Δ*SM*^	*Mypt1*^Δ*SM/*Δ*SM*^
Parent pairs	8	8	6
Litters	52	44^a^	2^b,c^
Total pups	288	246^a^	10^b,c^

^a^*Mypt1*^*+/*Δ*SM*^ mice showed significantly reduced litters and pups compared with *Mypt1*^*+/+*^ mice, *p* < 0.05;

^b^*Mypt1*^Δ*SM/*Δ*SM*^ mice showed significantly reduced litters and pups compared with *Mypt1*^*+/+*^ mice, *p* < 0.001;

^c^*Mypt1*^Δ*SM/*Δ*SM*^ mice showed significantly reduced litters and pups compared with *Mypt1*^*+/*Δ*SM*^ mice, *p* < 0.001.

**Table 2:** Summary of sexual behaviors of different genotyping mice*.

Behavior	Index	*Mypt1*^*+/+*^	*Mypt1*^*+/*Δ*SM*^	*Mypt1*^Δ*SM/*Δ*SM*^
	**No. of mice**	5/6	6/6	5/6
Sniffing	Frequency of tail sniff	5.60 ± 1.03	6.30 ± 1.12	4.60 ± 1.28
	Frequency of nose sniff	3.40 ± 1.03	3.50 ± 1.70	2.60 ± 0.68
Mounting	Frequency	3.20 ± 1.20	3.50 ± 0.94	4.60 ± 2.06
Grooming	Frequency	8.00 ± 1.41	8.80 ± 1.16	6.80 ± 1.72
Ejaculation	Frequency	0	0	0
Plug	**No. of mice**	6/6	6/6	6/6
	With a plug	6/6	5/6	0/6

^*^No tested behavior index except the presence of a vaginal plug was statistically significantly different among the three groups.

**Table 3:** Summary of sperm parameters of different genotyping mice (*n* = 9).

	*Mypt1*^*+ /+*^	*Mypt1*^*+/*Δ*SM*^	*Mypt1*^Δ*SM /ΔSM*^
Concentration (10^6^/ml)	5.26 ± 0.34	5.66 ± 0.22	5.94 ± 0.24
Motility %	35.08 ± 2.60	34.68 ± 2.51	35.50 ± 2.75

We then measured the penile ICP responses to EFS (electrical field stimulation) to evaluate the penile erectile function in mice. Upon treatment with an electric stimulus (5 V, 12 Hz, 1 ms pulse width for 60 s), the ICP/MAP of *Mypt1*^*+/+*^ mice increased rapidly and peaked at values of up to 0.50 ± 0.04 within 55 s; the ICP/MAP of *Mypt1*^*+/*Δ*SM*^ mice increased correspondingly, but the ratio (0.34 ± 0.03) was lower (*p* < 0.05). Strikingly, the ICP response to stimulation was abolished in *Mypt1*^Δ*SM/*Δ*SM*^ mice, and their ICP/MAP was significantly lower than that of the other groups (all *p* < 0.001) (Fig. 1a-b).

We also collected corpus cavernosum (CC) bodies from 5 PED-5i-invailed ED (the ED patients who had no beneficial response to PDE5 inhibitors over 2 years) and 3 penile carcinoma patients. MYPT1 expression from ED patients was significantly decreased by western blot, 2 of the 5 ED biopsies were even showed no detectable MYPT1 expression (Supplementary Fig. 3). Our collective observations showed that the ablation of MYPT1 led to ED.

### Ablation of MYPT1 expression impairs the contractility of penile smooth muscle

Histological examination showed that all penises of *Mypt1*^*+/+*^, *Mypt1*^*+/*Δ*SM*^ and *Mypt1*^Δ*SM/ΔSM*^ mice had comparable sizes and weights (all *p* > 0.05). Hematoxylin-eosin staining showed typical structures of the dorsal artery, dorsal vein, penile artery, CC body, tunica albuginea, urethra and sinus (Fig. 1c). Immunofluorescence staining showed intact fibrous fibers such as desmin and smooth muscle myosin heavy chain (SMHHC) (Supplementary Fig. 4). However, the diameter of the *Mypt1*^*+/*Δ*SM*^ and *Mypt1*^Δ*SM/*Δ*SM*^ dorsal artery was reduced significantly (*Mypt1*^*+/+*^: 98.90 ± 4.69 μm *vs Mypt1*^*+/*Δ*SM*^: 85.14 ± 2.59 μm, *p* < 0.05 *vs Mypt1*^Δ*SM/*Δ*SM*^: 58.94 ± 0.92 μm, *p* < 0.05) (Fig. 1d). As the relative area of the muscle layer was proportionally reduced (*Mypt1*^*+/+*^: 2283.00 ± 36.42 μm^2^ *vs Mypt1*^*+/*Δ*SM*^: 1782.00 ± 142.50 μm^2^ *vs Mypt1*^Δ*SM/*Δ*SM*^: 1299.00 ± 14.99 μm^2^) (Fig. 1e), the narrowed lumen was not likely to be caused by hypertrophy of the smooth muscle.

**Fig. 2:**
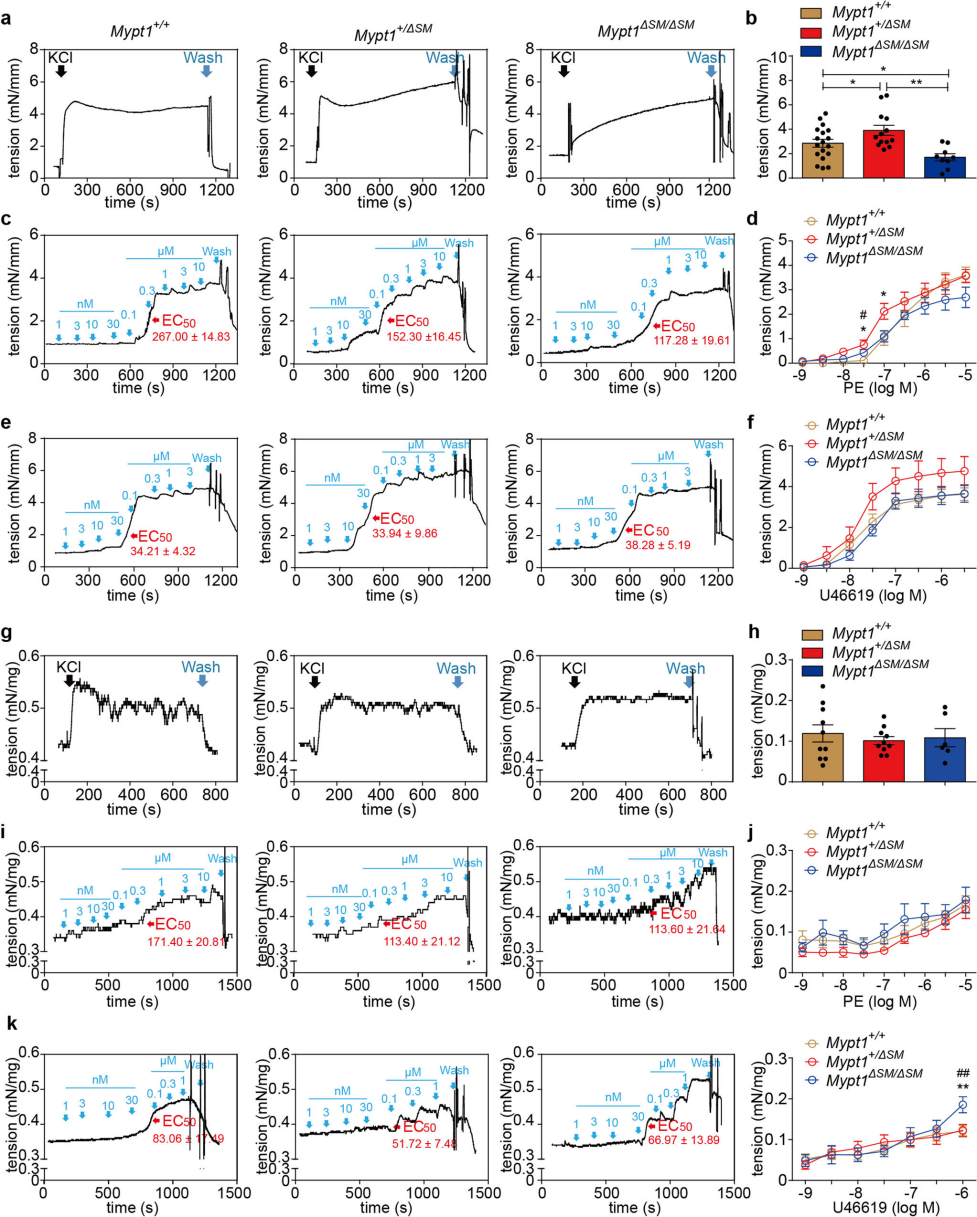
MYPT1-deficient dosal arteries and CCs showed increased sustained tension stimulated by KCl, and high sensitivity stimulated by agonists. **a, c** and **e**, Representative contraction of the dorsal arteries from *Mypt1*^*+/+*^, *Mypt1*^*+/*Δ*SM*^, and *Mypt1*^Δ*SM/*Δ*SM*^ mice in response to 124 mM KCl (**a**) and to increasing doses of PE (1 nM to 10 μM) (**c**) and U46619 (1 nM to 3 μM) (**e**). **b, d** and **f**, Quantification of the maximum tension force evoked by KCl (**b**) and the response sensitivity to PE (**d**) and U46619 (**f**) (Panel b: *Mypt1*^*+/+*^, *n* = 18; *Mypt1*^*+/*Δ*SM*^, *n* = 13; *Mypt1*^Δ*SM/*Δ*SM*^, n = 9; Panel d: *Mypt1*^*+/+*^, *n* = 5; *Mypt1*^*+/*Δ*SM*^, *n* = 6; *Mypt1*^Δ*SM/*Δ*SM*^, *n* = 4; Panel f: *Mypt1*^*+/+*^, *n* = 5; *Mypt1*^*+/*Δ*SM*^, *n* = 7; *Mypt1*^Δ*SM/*Δ*SM*^, *n* = 5). **g**, **i** and **k**, Representative tracings of the responses of CCs from *Mypt1*^*+/+*^, *Mypt1*^*+/*Δ*SM*^, and *Mypt1*^Δ*SM/*Δ*SM*^ mice evoked by 80 mM KCl (**g**) and to increasing doses of PE (1 nM to 10 μM) (**i**) and U46619 (1 nM to 3 μM) (**k**). **h, j** and **l**, Quantification of the maximum tension force evoked by KCl (**h**), and the response sensitivity to PE (**j**) and U46619 (**l**) (Panel h: *Mypt1*^*+/+*^, *n* = 10; *Mypt1*^*+/*Δ*SM*^, *n* = 10; *Mypt1*^Δ*SM/*Δ*SM*^, *n* = 6; Panel j: *Mypt1*^*+/+*^, *n* = 10; *Mypt1*^*+/*Δ*SM*^, *n* = 9; *Mypt1*^Δ*SM/*Δ*SM*^, *n* = 6; Panel l: *Mypt1*^*+/+*^, *n* = 7; *Mypt1*^*+/*Δ*SM*^, *n* = 10; *Mypt1*^Δ*SM/*Δ*SM*^, *n* = 6). The dose unit of EC50 is nM. The bars indicate the mean values ± SEM; *p < 0.05, **p < 0.01, ^#^*p* < 0.05; *One-way ANOVA*^.^ * represents the *P-value* between *Mypt1*^*+/+*^ and *Mypt1*^*+/*Δ*SM*^, ^#^ represents the *P value* between *Mypt1*^*+/*Δ*SM*^ and *Mypt1*^Δ*SM/*Δ*SM*^.

We then measured the contractile responses of the dorsal artery and CC muscles to KCl depolarization and GPCR agonists. Prior to regular measurements, we tested the intactness of endothelium of the dorsal artery by application of acetylcholine and observed that acetylcholine relaxed a PE-evoked preparation force efficiently (Supplementary Fig. 5), indicating the endothelium might be not impaired by our experimental manipulations. We also measured the endothelium layer by immune-staining with CD31 and confirmed the intactness of the endothelial layers both of control and MYPT1 knockout dorsal artieries (Supplementary Fig. 6). Note that, however, the effect of the nNOS on EFS stimulation remains to be determined. Upon depolarization by 124 mM KCl, the *Mypt1*^*+/*Δ*SM*^ artery displayed a 114.44~144.63% increase in the maximal force tension (*Mypt1*^*+/+*^: 2.85 ± 0.32 mN/mm *vs Mypt1*^*+/*Δ*SM*^: 3.91 ± 0.41 mN/mm *vs Mypt1*^Δ*SM/*Δ*SM*^: 1.70 ± 0.30 mN/mm) (Fig. 2a-b). We then assessed the response sensitivity of the MYPT1-deficient muscles to GPCR agonists, as shown in Fig. 2c–f. Interestingly, when treated with PE at concentrations as low as 3–30 nM, the MYPT1-deficient smooth muscles started to contract, while the control dorsal artery started to contract at doses as high as 100 nM (Fig. 2c-d). The force generated in the dorsal artery by 30 nM PE was 0.55 ± 0.15 mN/mm for *Mypt1*^*+/*Δ*SM*^ mice and 0.16 ± 0.04 mN/mm for *Mypt1*^Δ*SM/*Δ*SM*^ mice, all higher than that for control mice (*p* < 0.05) (Fig. 2c-d). Surprisingly, the initial responsive dose of PE in the dorsal artery was reduced approximately 10-fold when MYPT1 was ablated. The EC_50_ value of PE in dorsal arteries from homozygote were 152.30 ± 16.45 nM, which was also significantly lower than that in control arteries (267.00 ± 14.83 nM) (Fig. 2c, *p* < 0.001). This observation suggests that MYPT1-deficient dorsal arteries showed a hypersensitive response to PE, particularly at a low dose. U46619 did not enhance the contractile response in *Mypt1*^*+/*Δ*SM*^ and *Mypt1*^Δ*SM/*Δ*SM*^ dorsal arteries, and the response sensitivity was unchanged (Fig. 2e-f).

As CCSM is another important smooth muscle essential for penile erection, we then measured its contractile properties. Upon stimulation with 80 mM KCl, the peak force was not different among the *Mypt1*^*+/+*^, *Mypt1*^*+/*Δ*SM*^, and *Mypt1*^Δ*SM/*Δ*SM*^ groups (Fig. 2g-h). After stimulating with different doses of U46619, the evoked force of *Mypt1*^Δ*SM/*Δ*SM*^ muscle was significantly higher than that of *Mypt1*^*+/*Δ*SM*^ muscle (Fig. 2k-l), while it was comparable when stimulated with PE (*p* > 0.05) (Fig. 2i-j). Thus, CC smooth muscle displayed hyperresponsiveness to U46619 but not to PE. Because the mRNA of adrenergic receptor 1α (AR-1α) and adrenergic receptor 1β (AR-1β) were similar in the CC tissues between *Mypt1*^*+/+*^ and *Mypt1*^Δ*SM/*Δ*SM*^ (Supplementary Fig. 7), the different effects of MYPT1-deficient muscles may be not attributable to adrenergic receptors.

**Fig. 3:**
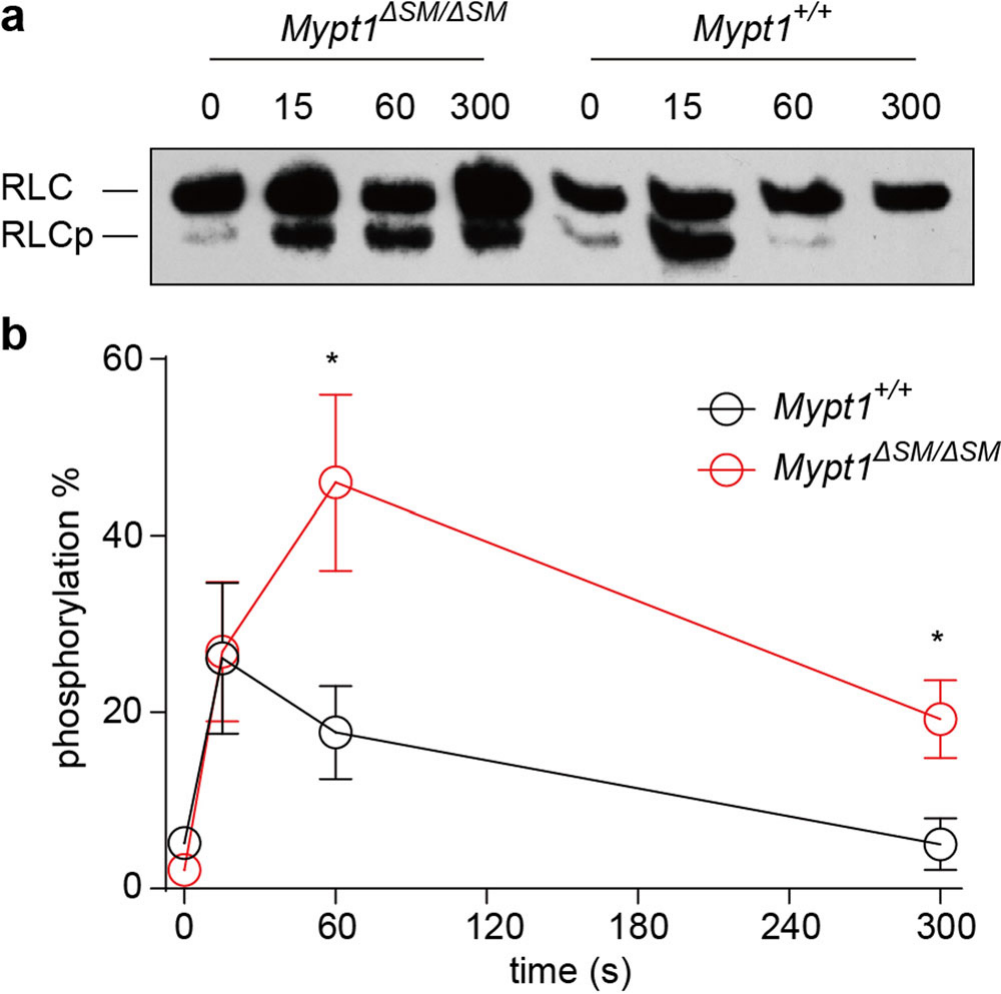
RLC phosphorylation in MYPT1-deficient dorsal arteries in response to PE stimulation. Dorsal arteries of MYPT1 KO (*Mypt1*^Δ*SM/*Δ*SM*^) and control (*Mypt1*^*+/+*^) mice were collected and stimulated with 10 μM PE. The stimulated arteries were sampled for RLC phosphorylation via urea/glycerol PAGE. **a** Representation of typical RLC phosphorylation assay results after stimulation. **b** Quantification of RLC phosphorylation. The RLCp level was expressed as the percentage of the total RLC level. The bars indicate the mean values ± SEM; *n* = 5; *p < 0.05; *t*-test.

RLC phosphorylation is tightly related to force development. To validate the hypothesis that the altered responses above were caused by natural force rather than by muscle remodeling, we measured RLC phosphorylation in the penile dorsal artery by urea/glycerol PAGE and Western blotting. As we expected, the induction of RLC mono-phosphorylation by PE was noticeably greater in *Mypt1*^Δ*SM/*Δ*SM*^ smooth muscle than in the control muscle (Fig. 3).

### Reduced MYPT1 expression impairs nitric oxide-mediated relaxation of penile smooth muscle

**Fig. 4:**
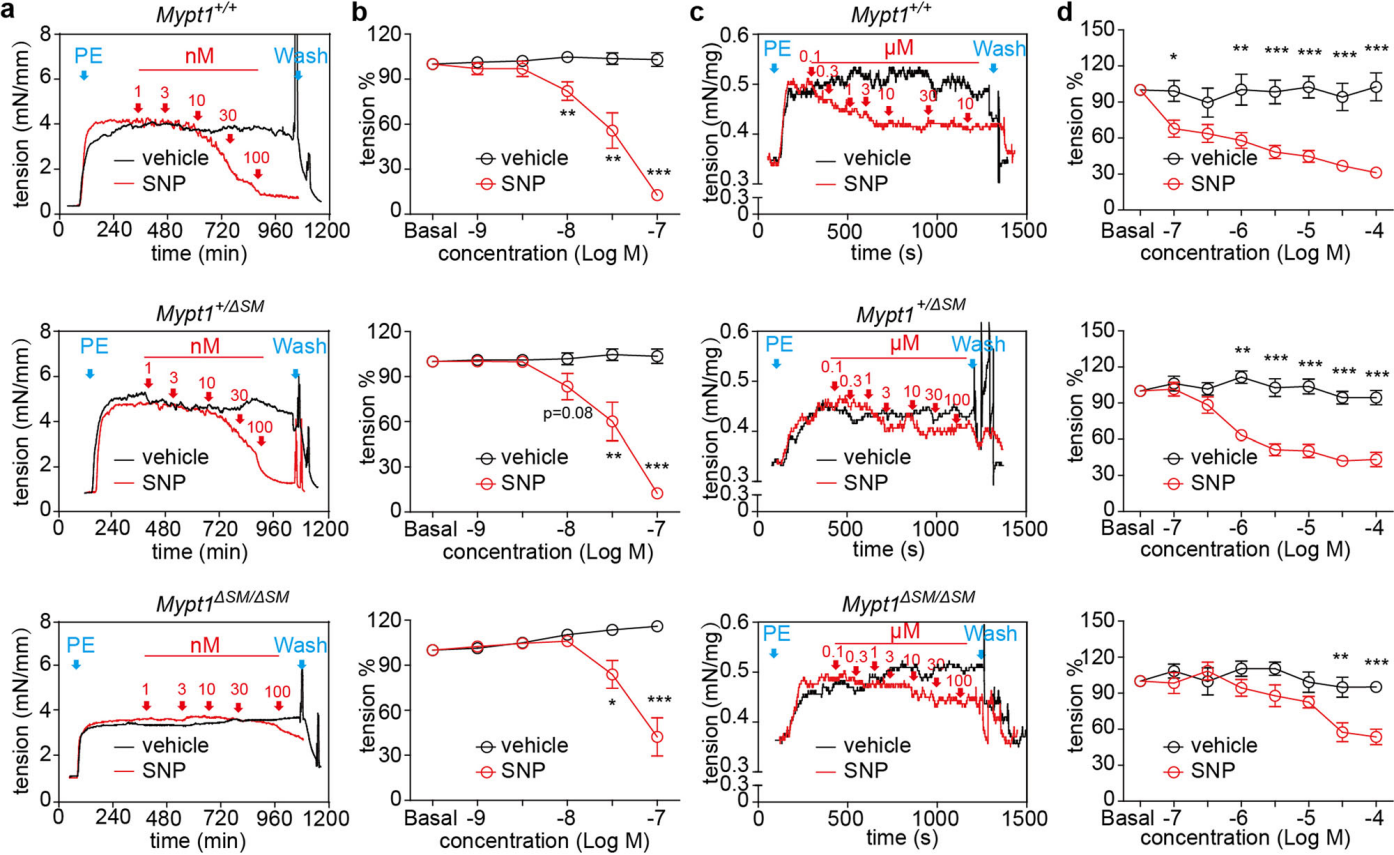
Relaxant effects of SNP on PE-evoked contraction of MYPT1-deficient dorsal arteries and CCs. **a** and **c**, Representative force tracings of dorsal arteries (**a**) or CCs (**c**) precontracted with 10 μM PE and then exposed to SNP (1 nM to 100 nM). **b** and **d**, Quantification of the relative ratios of force relaxation by the reagents (Panel **b**: *n* = 6; Panel **d**: *n* = 10). The relative ratio of the tension was calculated by the following formula: tension % = (F_vehicle_ – F_inhibitor_)/F_vehicle_, F = force. The bars indicate the mean values ± SEM; *p < 0.05, **p < 0.01, ***p < 0.001; *pair t-*test.

As activation of nitric oxide signaling is fundamental for erection, we then assessed the response of dorsal arteries to SNP, a nitric oxide donor. The PE-evoked force on the *Mypt1*^*+/+*^ and *Mypt1*^*+/*Δ*SM*^ dorsal arteries started to relax upon the addition of 10 nM SNP, but the effective dose was 30 nM for *Mypt1*^Δ*SM/*Δ*SM*^ dorsal arteries (Fig. 4a). This finding indicated that the deletion of MYPT1 led to a 3-fold increase in the SNP dose necessary to initiate relaxation. The respective SNP doses to induce 50% relaxation (EC_50_) in the *Mypt1*^*+/+*^, *Mypt1*^*+/*Δ*SM*^ and *Mypt1*^Δ*SM/*Δ*SM*^ groups were 26.94 ± 3.32 nM, 40.49 ± 6.63 nM and >100 nM or 185.60 ± 60.89 nM (calculated from fitting curve) (Fig. 4a-b, *p* < 0.01). This observation indicated an impairment of nitric oxide-mediated relaxation in the *Mypt1*^*+/*Δ*SM*^ and *Mypt1*^Δ*SM/ΔSM*^ dorsal artery. We also assessed the relaxation effect of ROCK (H1152) and PKC (GF109203) inhibitors. Relaxation of the *Mypt1*^*+/+*^, *Mypt1*^*+/*Δ*SM*^, and *Mypt1*^Δ*SM/*Δ*SM*^ dorsal arteries were reduced after the application of H1152 (Supplementary Fig. 8a-b) but not GF109203 (Supplementary Fig. 8c-d). In addition, similar relaxation effects were observed in CCSM. After treatment with SNP, CCSM form *Mypt1*^*+/+*^ started to relax at 30 nM, but the effective dose was 1 μM for *Mypt1*^*+/*Δ*SM*^ CCSM and 0.1 μM for *Mypt1*^Δ*SM/ΔSM*^ CCSM. Notably, 100 μM SNP relaxed the force by approximately 53.60%, 43.28%, and 31.23% in *Mypt1*^*+/+*^, *Mypt1*^*+/*Δ*SM*^, and *Mypt1*^Δ*SM/*Δ*SM*^ CCSM, respectively (Fig. 4c-d). The relaxation responses of *Mypt1*^*+/*Δ*SM*^ and *Mypt1*^Δ*SM/*Δ*SM*^ CCSM to H1152 and GF109203 were reduced (Supplementary Fig. 9). This effect indicated that the MYPT1 deletion impaired ROCK and PKC signal transduction. Meanwhile, compared with the *Mypt1*^*+/+*^, the *Mypt1*^Δ*SM/ΔSM*^ has the similar expression of AChR mRNA, eNOS and intact endothelial layer (Supplementary Fig. 6, 7). Our collective observations show impaired relaxation in MYPT1-deficient muscle, particularly in response to nitric oxide. Mechanistically, in penile smooth muscle, MYPT1 is required for cGMP/PKG/MYPT1/RLCp axis and ROCK/MYPT1^14^ and PKC/CPI-17/RLCp axis^17^. Whether the ablation of MYPT1 changed intracellular calcium during these processes remains to be determined.

### Pharmacological intervention with MYPT1 restores penile function in ED mice

**Fig. 5:**
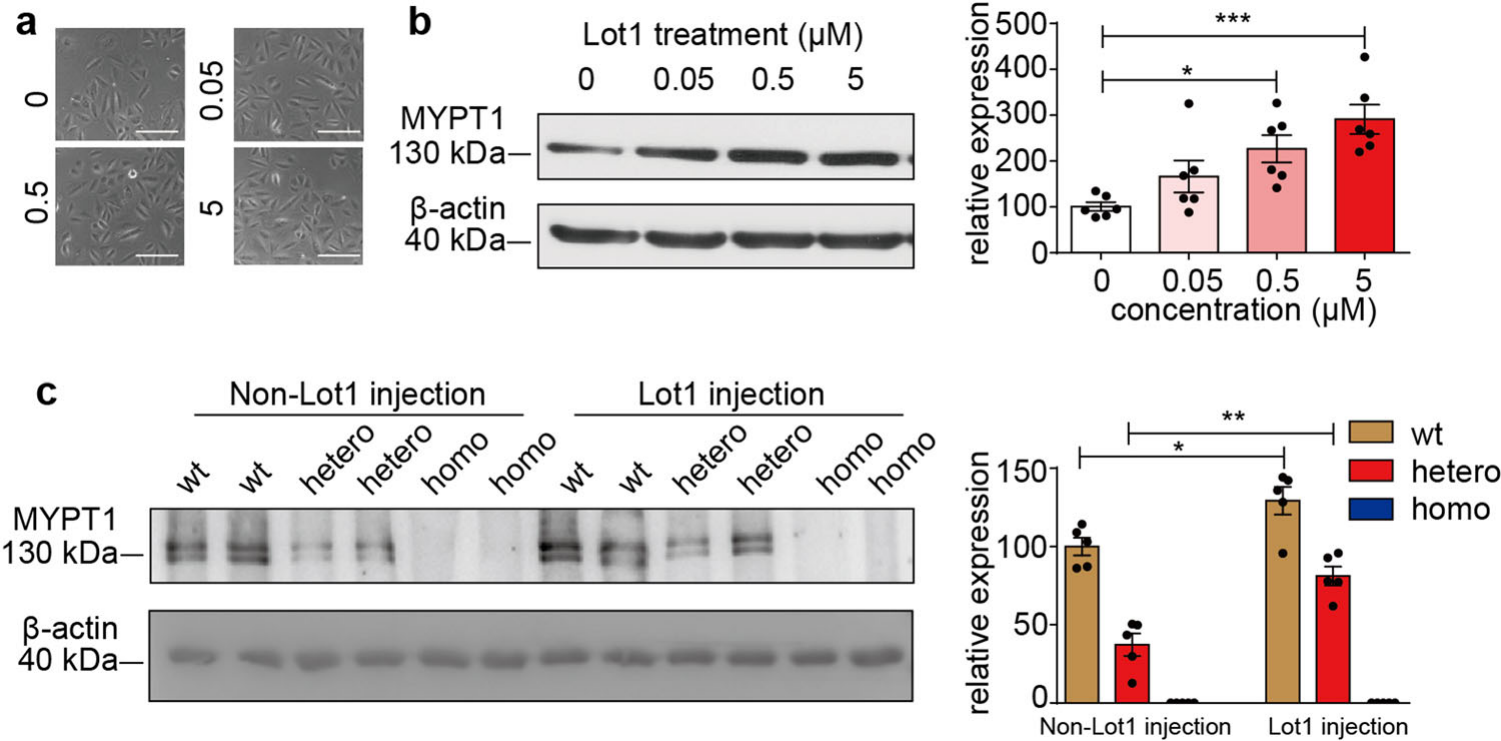
MYPT1 expression was increased by lotusine treatment both in vivo and in vitro. **a** A7R5 cells showed normal morphology after incubation with different doses of lotusine, which showed no cytotoxicity. **b** A7R5 cells were treated with lotusine in vitro for 24 h, and cell lysates were then subjected to Western blotting for MYPT1 measurement (*n* = 6) (*One-way ANOVA*). **c**, *Mypt1*^*+/+*^ *Mypt1*^*+/*Δ*SM*^ *Mypt1*^Δ*SM/*Δ*SM*^ mice were injected in vivo with lotusine (5 mg/kg), and MYPT1 protein expression in the penises was measured by Western blotting. Protein data were analyzed with β-actin as the internal control (*n* = 5) (*t-*test). The bars indicate the mean values ± SEM; *p < 0.05, **p < 0.01.

Given that a reduced MYPT1 level is essential for the ED phenotype, the upregulation of MYPT1 could be expected to restore penile function. We screened active compounds with potent activity towards, inducing MYPT1 expression. Briefly, we screened a compound library containing hundreds pure ingredients of traditional Chinese herbs (collected by our lab). Each compound was incubated with cultured smooth muscle cells (A7R5) for 48 hours, and then we measured MYPT1 protein by Western blot. After substaintially screening, we obtained some active compounds in which lotusine had relatively strong activity activity to upregulate MYPT1 level. In vitro, upon treatment with 0.5 µM Lot1, MYPT1 expression increased up to 2-fold, and increased further to about 3-fold while being treated with 5 µM Lot1 (Fig. 5a-b). After incubating A7R5 cells with Lot1 at this concentration for 24 h, the cell number and morphology had no apparent difference in contrast to the control cells, showing no apparent cytotoxicity. Based on the above effective concentrations of Lot1, we optimized and validated its in vivo effective dose. Lot1 was difficult to be absorbed by the gastrointestinal system, it was administrated by i.p. injection^21,22^. To optimize the proper dosage for MYPT1 expression, we i.p. injected Lot1 at dose of 5 mg/kg body weight every day and continued for 28 days. We found that Lot1 at dose of 5 mg/kg induced 1.3-fold elevation of MYPT1 expression in C57/BL6 mice and 2-fold in *Mypt1*^*+/*Δ*SM*^ mice (Fig. 5c) but did not increase in *Mypt1*^Δ*SM/*Δ*SM*^ mice. To avoid potential toxicity of lotusine at higher doses, we used the dosage of or less than 5 mg/kg body weight in our subsequent experiments.

**Fig. 6:**
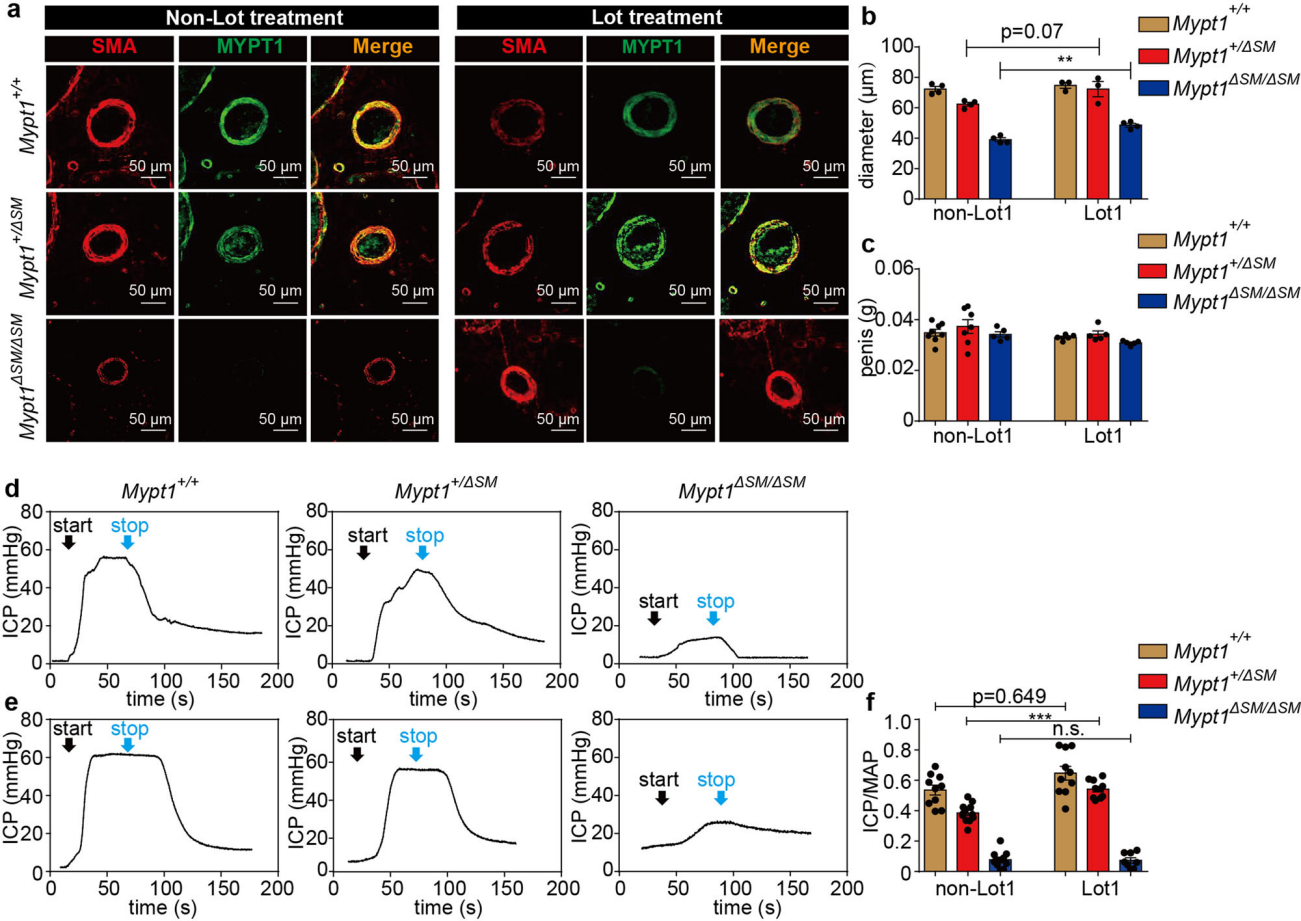
Lotusine improved the penile ICP response to EFS in MYPT1 haploinsufficiency mice. **a** Immunofluorescence staining showed an increase in the lumen sizes of the penile dorsal artery after lotusine treatment, and quantitation of the diameters is presented in panel **b**. SMA (red), smooth muscle actin, a specific marker of smooth muscle; MYPT1 (green) (Panel **b**: *Mypt1*^*+ /+*^, n = 4; *Mypt1*^*+ /ΔSM*^, *n* = 4; *Mypt1*^Δ*SM /ΔSM*^, *n* = 3). **c**, Quantitation of penis weights in the different groups of mice (non-Lot1: *Mypt1*^*+ /+*^, *n* = 8; *Mypt1*^*+ /ΔSM*^, n = 7; *Mypt1*^Δ*SM /ΔSM*^, *n* = 5; Lot1: *Mypt1*^*+ /+*^, *n* = 5; *Mypt1*^*+ /ΔSM*^, *n* = 5; *Mypt1*^Δ*SM /ΔSM*^, *n* = 5). **d**–**g**, MYPT1-deficient mice showed decreased ICP responses to EFS of the cavernous nerve. **d** and **e**, Representative tracings of ICP responses in *Mypt1*^*+/+*^, *Mypt1*^*+/*Δ*SM*^, and *Mypt1*^Δ*SM /ΔSM*^ mice without (**d**) or with lotusine treatment (**e**). **f** Quantification of ICP/MAP in the different groups of mice (non-Lot1: *Mypt1*^*+/+*^, *n* = 10; *Mypt1*^*+/*Δ*SM*^, *n* = 12; *Mypt1*^Δ*SM/*Δ*SM*^, *n* = 11; Lot1: *Mypt1*^*+/+*^, *n* = 10; *Mypt1*^*+/*Δ*SM*^, *n* = 10; *Mypt1*^Δ*SM/*Δ*SM*^, *n* = 8). The groups of Lot1 were injected by Lot1 (5 mg/kg body weight) once a day and continued for 28 days. The bars indicate the mean values ± SEM; **p < 0.001; ***p < 0.001; *t-test*.

As *Mypt1*^*+/*Δ*SM*^ mice showed a moderate ED phenotype (ICP/MAP = 0.34 ± 0.03) (Fig. 1a), we used these mice as an ED disease model, although the penile function was not impaired enough to affect fertility. We treated these mice and their littermates with Lot1 via consecutive *i.p*. injections. The Lot1-treated *Mypt1*^*+/*Δ*SM*^ and *Mypt1*^Δ*SM/*Δ*SM*^ penises showed sizes comparable to those of *Mypt1*^*+/+*^ penises (Fig. 6c). Surprisingly, the lumen diameter of the *Mypt1*^*+/*Δ*SM*^ and *Mypt1*^Δ*SM/*Δ*SM*^ dorsal artery and CC was also restored significantly (Fig. 6a-b, Supplementary Fig. 10). We then measured the penile ICP/MAP in response to stimulation. Upon stimulation with 5 V/12 Hz, control penises treated with Lot1 displayed a 110% increase in the ICP/MAP across the untreated group, but the difference was not statistically significant (*p* = 0.65) (Fig. 6d, e and f). However, *Mypt1*^*+/*Δ*SM*^ penises showed a significant increase of ICP/MAP after Lot1 treatment (treatment: 0.54 ± 0.02 *vs* no treatment: 0.39 ± 0.02, *p* < 0.001), comparable to the effect in *Mypt1*^Δ*SM/*Δ*SM*^ mice (*p* > 0.05) (Fig. 6d, e and f). Collectively, these observations showed that treatment with Lot1 significantly restored penile function through the upregulation of MYPT1. As Lot1 treatment did not restore penile function in *Mypt1*^Δ*SM/*Δ*SM*^ mice, in which both *Mypt1* alleles had been deleted, the efficacy of Lot1 treatment was primarily mediated by targeting MYPT1.

### MYPT1 protein expression can regulate penile erection in db/db mice

Diabetes is a risk factor in ED genesis that usually is insensitive to PDE5 inhibitor, and diabetic (db/db) mice is usually considered to be an ED mice model^3^. We measured the ICP of db/db mice in response to sildenafil (2 mg/kg, *i.v*., 10 min), a widely used inhibitor of PDE5, and found these mice had a much weak response in contrast to BKS control mice (Supplementary Fig. 11a-b). These diabetic mice appear to be a good ED model featured by low or non-reposnive to sildenafil treatment. We also detected the expression of eNOS and MYPT1 in the dorsal artery and CC by immunofluorescence staining, the results showed eNOS has slightly decreased in db/db mice (Supplementary Fig. 11d-e), while the MYPT1 protein was significantly reduced (Supplementary Fig. 11c and e). This result showed that in the diabetic ED mice model, the decreased MYPT1 existed.

**Fig. 7:**
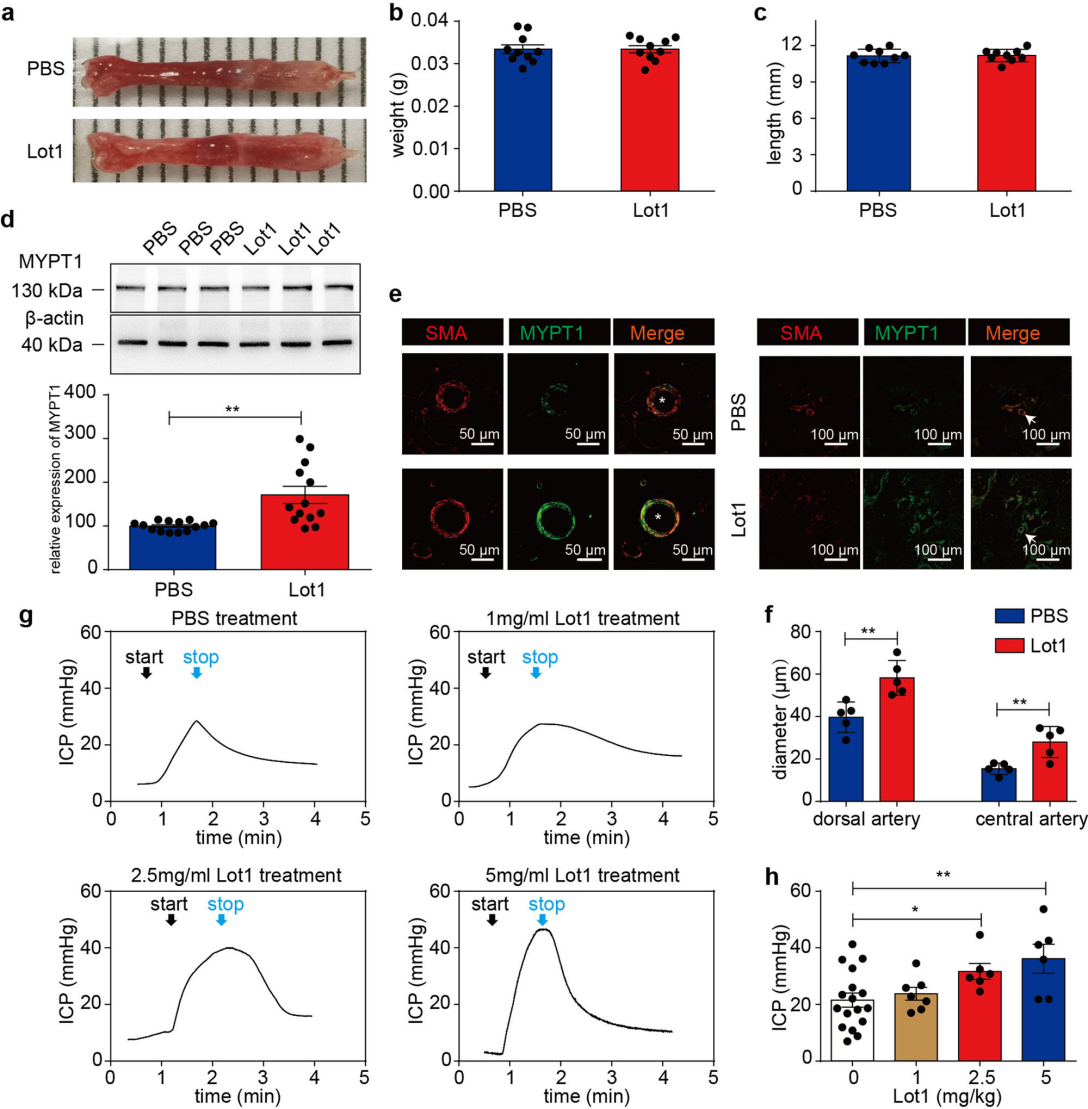
Lotusine improved the ICP response to electric stimulation in *db/db* mice. **a**–**c** The penile weights and lengths were unchanged after lotusine treatment (Panel **b**, *n* = 10; Panel **c**, *n* = 9) (*t-test*). **d** Western blot analysis showed increased in MYPT1 expression in penis after Lot1 treatment (PBS, *n* = 14, Lot1, *n* = 13) (*Welch’s t-test*). **e** Immunofluorescence staining showed an increase in penile MYPT1 expression after Lot1 treatment. **f**, Quantitation of the diameters is presented in panel. (*n* = 5) (*t-test*). **g** Representative ICP tracings in *db/db* mice with (0, 1, 2.5 or 5 mg/kg) lotusine treatment. **h**, Quantification of the ICP values in the different groups of mice (0 mg/kg, *n* = 17; 1 mg/kg, *n* = 7; 2.5 mg/kg, *n* = 6; 5 mg/kg, *n* = 6) (*One-way ANOVA*). The bars indicate the mean values ± SEM; *p < 0.05; **p < 0.05. ‘Lot1’ indicates the group that received lotusine treatment. “asterisk” represents dorsal artery; “arrow” represents CC.

40-60% of patients failed with PED-5Is in certain disease states, especially in diabetic ED^23–25^. We treated *db/db* mice with 5 mg/kg Lot1 for 28 days and found that MYPT1 expression in dorsal artery was upregulated 1.5-fold compared with that in PBS-treated mice (Fig. 7d), while about 2-fold for aorta (Supplementary Fig. 12). Treated mice showed penis sizes comparable to those of control mice (Fig. 7a–c) but enlarged artery lumens (Fig. 7e-f). Meanwhile, the ICP was increased by about 47.04% after treatment with 2.5 mg/kg Lot1, and was increased by about 89.99% with 5 mg/kg treatment compared with PBS-treatment. (Fig. 7g-h). These results showed that Lot1 improved the erectile function and penile artery diameter of ED mice with a dose-dependent effect. These results showed that Lot1 can rescued the ED phenotype in diabetic mice. However, more grouped animals are necessarily required for assess this effect substaintially in the future.

### Lotusine induces an increase in MYPT1 expression by inhibiting the ubiquitin pathway

**Fig. 8:**
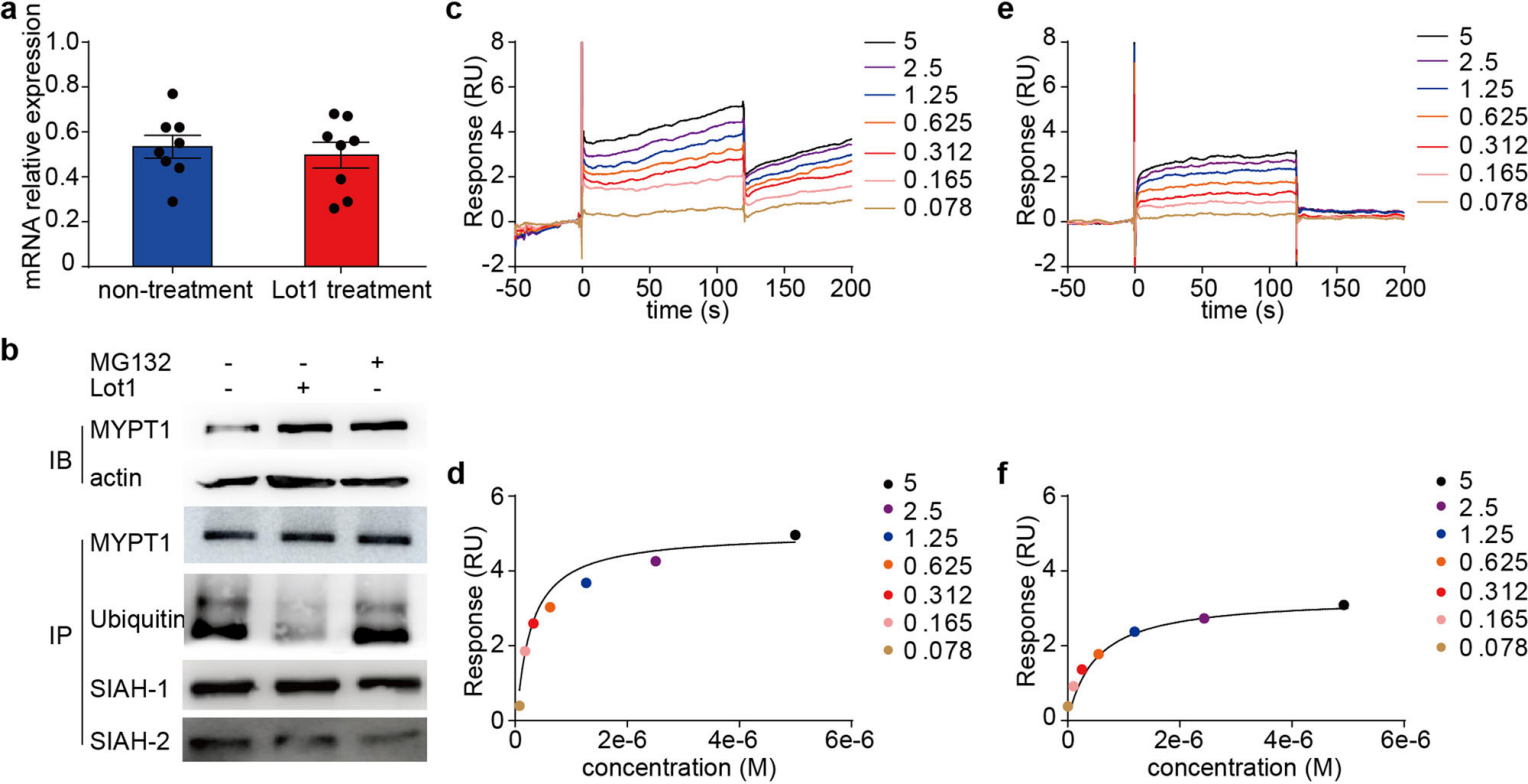
Lotusine upregulated MYPT1 expression by inhibiting MYPT1 ubiquitination by binding SIAH1/2. **a** *Mypt1* mRNA levels in A7R5 cells treated with or without lotusine were unchanged, as measured by Q-PCR (*n* = 8). **b**, A7R5 cells were treated with MG132 (20 μm, 4 h) and lotusine (0.5 μm, 24 h), and the lysates were subjected to immunoprecipitation (IP) with an anti-MYPT1 antibody and immunoblotting (IB) for ubiquitinated MYPT1, SIAH2 and SIAH2 (*n* = 3) in the immunoprecipitate. c-e, Representative binding sensorgrams showing the kinetics of Lot1 binding to SIAH1-GST (**c**) and SIAH2-GST (**e**). **d**–**f**, Quantification of the kinetics of Lot1 with SIAH1-GST (**d**) and SIAH2-GST (**f**). The bars indicate the mean values ± SEM; Lot1=lotusine.

To investigate the regulatory mechanism of Lot1, we compared *Mypt1* mRNA levels in A7R5 cells treated with and without Lot1. Treatment with 0.5 μm Lot1 did not affect the *Mypt1* mRNA level (p > 0.05) (Fig. 8a), suggesting that a posttranslational pathway regulates the MYPT1 protein level. We then used an anti-MYPT1 antibody to pull down MYPT1 protein from lysates of A7R5 cells treated with 20 μM MG132 (an proteasome inhibitor) and 0.5 μM Lot1 for 24 h and subjected the precipitated mixture to immunoblotting to detect ubiquitin. The level of total MYPT1 was elevated after respective treatment with MG132 and Lot1, while the level of ubiquitinated MYPT1 treated with Lot1 was decreased (Fig. 8b). In addition, both the SIAH1 and SIAH2 E3 ligases, which contain an MYPT1 binding motif, were detected in the immunoprecipitated (Fig. 8b). To test if Lot1 directly binds to SIAH1/2 E3 ligases, we expressed recombinant GST-SIAH1 and GST-SIAH2 proteins in bacteria and subjected these proteins to binding affinity assay with a Biacore system. The result showed that Lot1 could directly bind the recombinant proteins with high affinities (SIAH1: K_D_ = 2.4e-7 and SIAH2: K_D_ = 5.03e-7) (Fig. 8c–f). Together, these results showed that lotusine upregulated MYPT1 protein through directly binding SIAH1 and SIAH2.

## Discussion

ED is a worldwide disease which affects millions of people, and a normal penile erection is even the first step in fertility. The robust erectile function ultimately relied on the penile smooth muscle contraction. Many researches have reported that multiple factors can affect this process, such as endothelial dysfunction^26^, cGMP deficiency^27^, reduced PKG1 activity^26^, elevated RhoA and ROCK activity^26,28^ and so on. However, the target tissues all these factors point to smooth muscle. For example, although the endothelial dysfunction is indeed important for ED, in the context of erectile, the smooth muscle is a critical target of endothelium. In this report, we indeed found that downregulation of MYPT1 significantly altered the contractile properties of penile smooth muscles, and was sufficient to cause serious ED phenotypes and histological changes. On the other side, the upregulation of MYPT1 not only improved penile erection but also restored tissue structures. As MYPT1 expression has been proved to be efficiently regulated by pathological stimuli^29^, our present results thus revealed a novel pathogenic mechanism for vasculogenic ED. In constrast to current proposal that endothelial dysfunction caused by CVD serves as a factor of vascularogenic ED^4,5,30,31^, our proposal emphasizes the importance of smooth muscle contractile behaviors critically mediated by MYPT1.

As eNOS expression is not regularly reduced in penile tissues both of ED patients and db/db mice as we observed, the reduction of MYPT1 protein may be a leading mechanism linking ED and CVD. Lotusine is an alkaloid from Nelumbo nucifera^32^, and the soluble lotusine alkaloid has been discovered long time before. Current observations suggest that lotusine benefits cardiovascular, intestinal and tracheal diseases^33,34^. There are reports showing that lotusine targets on L-type calcium channel^35^ and oxidative stress in cardimyocytes^32^, but the regulatory-specific mechanism remains to be determined. Our observation reported here suggests that lotusine efficiently targets on MYPT1 expression in penile smooth muscle and may serve as a prospective candidate for ED therapy. As lotusine also elevates MYPT1 expression of vascular smooth muscle cells, it is expected that it will improve the functions of the cardiovascular system simultaneously.

Based on our observations, besides the imbalance in NO/cGMP/PKG pathway^7–9^, the ED genesis may also occur via the following scenario: pathological stimulation induces downregulation of MYPT1 in penile smooth muscle; the reduction in MYPT1 leads to enhanced contractile responses to agonists and resistance to nitric oxide; the resultant hypercontractility inhibits the blood filling process, and the penis adopts a persistent flaccid state, and resistance to nitric oxide inhibits the vessel dilation process. As blood filling is required not only for erection but also for the penile nutrient supply, the arterial narrowing caused by the persistent flaccid state further limits blood filling and hence an erection. The interplay of these regulatory processes appears to contribute heavily to ED genesis.

MYPT1 is encoded by the 4.6 kb *Ppp1r12a* gene and is ubiquitously and constantly expressed as a housekeeping gene^36^. The MYPT1 protein contains SIAH1/2 E3 ligase binding motifs, implying the involvement of a ubiquitin-E3 ligase-proteasomal degradation pathway in MYPT1 regulation^37^. Our recent report validated this pathway in colonic smooth muscle^29^. In this report, we demonstrated that this pathway also exists in penile smooth muscle. Interestingly, miRNA and several pathological factors (e.g. LPS and high glucose) can regulate MYPT1 expression^38–42^. Decreased MYPT1 expression was detected in gastric fundus and gastric antrum smooth muscle from diabetes patients^43^ and ob/ob mice^44^, moreover the activity of SIAH1 was increased in high glucose environment^45^. This implies the involvement of MYPT1 in the ED phenotype complicated with other disorders such as diabetes^3,8,46^ or bowel inflammation^47^.

Nitric oxide in penile tissue is released from nerves or synthesized by eNOS and mediates smooth muscle relaxation by stimulating guanylyl cyclase (GC) activity towards cGMP production, thereby mediating relaxation through cGMP-dependent kinase (PKGIα)^48,49^. PDE5 inhibition amplifies nitric oxide-cGMP signaling by overproducing cGMP, resulting in enhanced relaxation^48^. PDE5 inhibitors are currently used for first-line treatment of ED, and several new selective inhibitors are in development. However, according to current clinical results, at least 30% of ED patients do not respond to PDE5 inhibitors, in diabetic ED patients, the failed-treated patients were up to 40%-60%^3^. Meanwhile, the eNOS in the diabetic animal model did not significantly decrease in CC^50,51^. We here found that the ablation of MYPT1 expression led to reduced sensitivity to nitric oxide for penile smooth muscle relaxation. This effect may result from the abolition of PKGI-MYPT1-Ser695 signaling during relaxation^16,19^ and, interestingly, might partially explain the therapeutic failure of PDE5 inhibitors. Enhance the expression of MYPT1 by lotusine or inhibit the SIAH1/2 E3 ligase-proteasomal MYPT1 degradation by local injection of adenovirus might help as a treatment to improve ED.

Relaxation of penile smooth muscles (artery and CC) is the final goal for ED therapy, and there are two basic strategies so far. The first one is releasing smooth muscle contraction by declining intracellular calcium concentration elevated by membrane depolarization-mediated calcium influx and GPCR-mediated calcium release from SR. For this strategy, L-type calcium channel blocker, GPCR antagonists, and KCa openers for hyperpolarizing endothelial and smooth muscle cells and down-regulators KCa for endothelial cells have been proposed for ED therapy^52^. Another strategy is directly initiating smooth muscle relaxation by dephosphorylating myosin light chain via calcium sensitization mechanism. For this strategy, ROCK inhibitors and PDE5 inhibitors have been designed although these inhibitors might also affect intracellular calcium^53^. However, the most successful drugs of ED belong to PDE5 inhibitors. In addition, there is evidence showing that endothelial KCa activation is able to enhance sildenafil-induced relaxation in penile artery and thereby restores sildenafil sensitivity of diabetic ED^54^. This implies that the combination with different therapeutic strategies might be more effective. In this report, the reduction of MYPT1 not only impaired relaxation of NO/pKG signaling but also enhanced calcium sensitized contraction of penile smooth muscle. This suggests that up-regulation of MYPT1 would be expected to simultaneously restore the abnormal relaxation/contraction properties and hence possibly will exhibit therapeutic efficacy. In the future studies, it would be also interesting to combine the therapy of MYPT1 up-regulation with other therapy strategies including calcium declining (e.g. K^+^ or Ca^2+^ channel modulators).

As MLCP is able to target several signal modules, the ablation of MYPT1 potentially affects other erection-associated processes beyond smooth muscle contractility. We had not examined these processes of the MYPT1-deficient penile tissues in this report, such as the dynamic alteration of intracellular calcium; the nitric oxide response to EFS. Moreover, further measurements of MYPT1 phosphorylation and RLC phosphorylation of lotusine-treated penile smooth muscle will enhance our understanding for ED genesis. Applying more animals for the experimental groups would also solitude our conclusion further.

## Materials and methods

### Animals

*Mypt1*^*+/+*^, *Mypt1*^*+/*Δ*SM*^ and *Mypt1*^Δ*SM/*Δ*SM*^ mice were generated previously^16^, and db/db mice were bought from the Model Animal Research Center of Nanjing University. All the mice were maintained at the Model Animal Research Center of Nanjing University, and the male mice were used at 8-14 weeks. The 8-10 weeks male mice were used in male sexual behavior test and Lot1 injection. For the experiments such as sperm quality test, ICP, PDA smooth muscle contractility, CC contractility, HE and immunofluorescence, the male mice were sacrificed at 10-14 weeks. Within the same experiment, the age of the grouped mice were similar. The db/db mice were purchased at 8-10 weeks and sacrificed at 12-14 weeks after treatment. All the diebetic mice are quality controlled (by the animal vendor) by measuring body weight, 6 h fasting blood glucose, HbA1c and insulin when acquired the BKS-db mice. At 12-weeks-old, the quality data are: BKS-db *vs* BKS, weight: 45 g *vs* 25 g; 6 h fasting blood glucose: 33 mmol/L *vs* 9 mmol/L; HbA1c: 8.6% *vs* 4%; insulin: 16 ng/ml *vs* 5 ng/ml. The animal experiments performed in this study were conducted in accordance with the guidelines of the Animal Care and Use Committee of the Model Animal Research Center of Nanjing University (Nanjing, China) (#ZMS-24). All applicable institutional and/or national guidelines for the care and use of animals were followed.

### Clinical biopsies

Human CC tissues were collected from ED patients undergoing penile prosthesis implantation surgery at Peijing Hospital. The normal CC tissues adjacent to the tumors were collected from the patients with penile carcinoma patients at Nanjing Jinling Hospital. Eligible participants were men aged from 22 to 62-year-old. The ED patients were diagnosed by the International Index of Erectile Function (IIEF) and Audiovisual Sexual Stimulation (AVSS). The average IIEF score was ≤8, and the AVSS was negative. The patients had persistent ED phenotypes at least 7 years and had no beneficial response to PDE5 inhibitors over 2 years. The IIEF score was ≥20 in carcinoma patients. All the experiments were approved by the Research Ethics Committee of Beijing Hospital and Research Ethics Committee of Nanjing Jinling Hospital (2020DZGZRZX-094), and all patients provided written informed consent.

### Male sexual behavior test

Sexual behavior was tested as described in a previous report^55^. A male (8–10 weeks) and a female (6–8 weeks) mouse were manually placed in the same chamber, and their sexual behaviors were visually assessed at 8:00 pm over a 45-min measurement period. Before behavior recording, the female mice were hormonally primed for 48 hours via subcutaneous injection of 10 μg of estradiol benzoate (Sigma-Aldrich) and were then injected with 500 μg of progesterone (Sigma-Aldrich) at 3-5 hours before recording. Both reagents were dissolved in sesame oil. The plug in the vaginal orifice was observed at 9:00 am on the second day.

### Sperm quality test

The cauda epididymis was incised and dissected in 1 ml prewarm human tubal fluid (HTF) medium (Easycheck Cat. No. M1130). After equilibration at 37 °C for 15 min, the sperm quality, including sperm concentration and activity was assessed by the Computer Assisted Semen Analysis (CASA) system. 10 μl suspension was used for sperm smears, which was fixed by methyl alcohol for 15 min, then stained by 2% eosin for 2 h. The morphology of the sperm was observed by microscope (Olympus).

### In vivo measurements of intracavernous pressure (ICP) and mean arterial pressure (MAP)

To evaluate the penile erectile function of mice, the ICP in response to electrostimulation of cavernous nerves was measured according to a previous report with modification^56,57^. Mice were anesthetized by intraperitoneal injection of pentobarbital (40 mg/kg), and the bladder and prostate were exposed via a midline suprapubic incision. The testes and epididymides were repositioned into the abdomen to expose cavernous nerves. The penis was denuded of skin and fascia, and a heparinized (100 IE/mL) 25-gauge needle was inserted into the CC and connected to a pressure transducer via polyethylene (PE)-50 tubing. The catheter was connected to a pressure transducer connected to a PowerLab 8/SP data acquisition system (Chart 5.0 software; ADInstruments, Colorado Springs, Australia). The parameters for electric stimulation of cavernous nerves were as follows: 5 V, 12 Hz, 1 ms pulse width, and 60 s duration. MAP was measured as previously reported (ALC Non-invasive Blood Pressure System, Shanghai Alcott Biotech, China)^19^.

### Measurement of the penile dorsal artery (PDA) smooth muscle and CCSM contractility

The cavernosal strips with albuginea were isolated from the base of the penis and the dorsal penile arteries were dissected from the appendix tissue. The fresh arteries were cut off with 1.4-mm length. The segment was threaded onto two steel wires and then mounted in a small-vessel wire myograph chamber (610-M; Danish Myo Technology, Aarhus, Denmark) containing Krebs solution (NaCl, 130 mM; NaHCO_3_, 14.9 mM; dextrose, 5.5 mM; KCl, 4.7 mM; KH_2_PO_4_, 1.18 mM; MgSO_4_·7H_2_O, 1.17 mM; and CaCl_2_·2H_2_O, 1.6 mM) that was continuously aerated with 95% O_2_/5% CO_2_. After adjusting the resting tension to a value equal to 100 mm Hg in vivo^58^, the mounted artery segment was equilibrated for an additional 20 min before treatment with 124 mM KCl Krebs solution (NaCl, 10.7 mM; NaHCO_3_, 14.9 mM; dextrose, 5.5 mM; KCl, 124 mM; KH_2_PO_4_, 1.18 mM; MgSO_4_·7H_2_O, 1.17 mM; and CaCl_2_·2H_2_O, 1.6 mM) or other reagents. PE and U46619 were used as GPCR agonists for evoking smooth muscle contraction; H1152 was used as a ROCK inhibitor, and GF109203x was used as a PKC inhibitor. To measure CCSM contractility, the intact tunica albuginea was isolated from the penis in prechilled Krebs solution and cavernosal strips were then mounted to isometric transducers MLT0201 (ADInstruments) and equilibrated in Krebs solution at 37 ^°^C for 30 min. When we applied the different animal models for force measurement, the basal tension should be reset individually. Considering the prolonged manipulation for tension adjustment possibly impaired contractile response, we only tested the basal tension of 0.2 g and 0.5 g. We observed that the basal tension around 0.5 g was good for producing maximal force tension for those groups of animals. To simplify the measurement protocol, we used 0.5 g as the single basal tension in our subsequent experiments. After adjustment for rest tension at 0.5 g^59^, the cavernosal strips were stimulated with 80 mM KCl Krebs solution (NaCl, 55.5 mM; NaHCO_3_, 14.9 mM; dextrose, 80 mM; KCl, 4.7 mM; KH_2_PO_4_, 1.18 mM; MgSO_4_·7H_2_O, 1.17 mM; and CaCl_2_·2H_2_O, 1.6 mM) or GPCR agonists. The tension of DAs and CCs were recorded using a PowerLab/8SP data acquisition system (Chart 5.0 software; ADInstruments, Colorado Springs, Australia).

### Reagents administration

Lotusine powder is dissolved in PBS and PBS is used as vehicle control. Different doses of lotusine (1, 2.5 or 5.0 mg/kg body weight) were *i.p*. injected to male mice (age: 8-10 weeks) once a day and continued for 28 days. The therapeutic effectiveness is then assessed by measurement of ICP and histology.

Sildenafil (PHR1807, sigma) is dissolved in PBS and PBS is used as vehicle control. Prior to measuring ICP, sildenafil (2 mg/kg, *i.v*.) is intravenously injected to each mouse.

### Immunofluorescence

Penises were fixed with 4% formaldehyde supplemented with 0.002% picric acid for 4 hours at room temperature and were then dehydrated in 30% sucrose PBS overnight at 4 ^°^C. Fixed tissues were embedded in optimal cutting temperature (OCT) compound (Leica) and cut into 10-μm sections. Sections were blocked with 0.1% Triton X-100/0.1% Tween 20/3% nonimmune goat serum in PBS for 1 hour at room temperature Sections were incubated with primary antibodies (anti-MYPT1 (1:200, Cat. No. 22117-1-AP, Proteintech); anti-smooth muscle α-actin (1:200, Cat. No. ab7817, Abcam); anti-eNOS (1:200, Cat. BS3571, BioRad); antidesmin (1:200, Cat. No. 21404-1-AP, Proteintech); anti-SMHHC (1:200, Cat. No. 16520-1-AP, Proteintech), anti-CD31 (1:200, Cat.No.550274, BD Biosciences) overnight at 4 ^°^C and then with fluorescent secondary antibodies (Invitrogen) for 2 hours at room temperature. Fluorescence staining was examined under a confocal microscope (Olympus).

### Histological examination of mouse penises

Mouse penises and testis were fixed in 4% formaldehyde at 4 °C for 2 h, dehydrated in butyl alcohol at 4 °C overnight, embedded in paraffin, and cut into 5-μm sections. Sections were stained with hematoxylin/eosin (H-E), the morphology of penile arteries was examined via microscopic imaging (DotSlide, Olympus).

### Western blot analysis

To evaluate contractile-related proteins, CCs from patients were homogenized in lysis buffer (2% SDS; 10% glycerol; 10 mM DTT; and 50 mM Tris-HCl (pH 7.4)). After incubation at 85 °C for 5 min, the samples were centrifuged at 10,600× *g* for 10 min^60^. The protein concentrations were measured with bicinchoninic acid protein assay reagent (Thermo Scientific Pierce). Equal amounts of protein were separated by 8-12% SDS-PAGE, and the proteins were then transferred to a PVDF membrane. After blocking by 5% non-fatted milk, the membrane was sequentially probed with a primary antibody and a secondary antibody. To visualize the immunoreaction signal, the membrane was incubated in Subpico Western Solution (Sudgen) and exposed to film. The following primary antibodies were used for Western blotting: anti-smooth muscle β-actin (IB: 1:10,000, Cat. No. A5441, Sigma), anti-MYPT-1 (IP: 1:50; IB: 1:2000, Cat. No. 22117-1-AP, Proteintech), anti-RLC^61^.

### Measurement of myosin light chain phosphorylation^61^

Myosin light chain phosphorylation in the penile artery was measured via urea/glycerol PAGE. Briefly, after stimulation with 10 μM PE, the artery was immediately frozen in liquid nitrogen at the indicated time point and stored in a solution of 10% TCA/10 mM DTT in acetone at -80 ^°^C. The tissues were then ground to a slurry with 10% TCA/10 mM DTT in H_2_O. The mixtures were centrifuged at 3,000 × g for 3 min, and then the supernatants were discarded. The precipitates were washed 2 times with acetone and one time with diethyl ether, dried at room temperature after removal of the diethyl ether and dissolved in 8 M urea sample buffer. An anti-RLC antibody was used as the primary antibody for Western blotting. The relative ratio of phosphorylated RLC to total RLC was calculated.

### Preparation of recombinant SIAH1 and SIAH2 proteins

Mouse *Siah1 and Siah2* cDNAs were amplified from smooth muscle tissue by RT-PCR kit (Invitrogen) and confirmed by DNA sequencing. The coding fragments were respectively inserted into a pGEX-5x-1 vector *via Bam*H I/*EcoR*I polyclonal restriction sites, and then transformed the recombinant plasmids to BL21 E.Coli. After induction with 1 mM isopropyl-β-d-thiogalactopyranoside (IPTG), the bacteria successfully expressed recombinant GST-SIAH1 and GST-SIAH2 proteins. We purified these proteins with a glutathione sepharose resin (Cat. 17-0756-01, GE Healthcare) and the purity of these proteins was higher than 95%.

### Binding assay for lotusine and recombinant SIAH1 and SIAH2 proteins

The respective binding affinities of lotusine with SIAH1 and SIAH2 were determined by a Biacore T200 instrument. The SIAH1-GST and SIAH2-GST protein (with the concentration of 20 μg/ml) were respectively immobilized on a CM5 chip at pH 4.0 by using the amine coupling kit (GE Healthcare). A dilution series of lotusine (0 to 5 μM) was passed on SIAH1-GST and SIAH2-GST at 20 μl/min and the association phases (120 s) were recorded. The binding kinetics were analyzed with a 1:1 binding model of Biocore evaluation software. The kinetic rate constants, Ka, and Kd were derived for each reaction. K_D_ values were calculated by Kd/Ka ratio.

### Statistics and Reproducibility

Data are presented as means ± SEM. Differences between two groups were evaluated by paired or unpaired t-tests. When there was heterogeneity of variance according to the *Levene’s test*, we used the *Welch’s t-test*. Multiple group comparisons were performed by using *one-way ANOVA* followed by Tukey’s test. *P* ≤ 0.05 was considered statistically significant. All statistical analyses were performed in SPSS 20.0 software.

### Reporting summary

Further information on research design is available in the Nature Research Reporting Summary linked to this article.

## Supplementary information

Supplementary Information

Description of Additional Supplementary Files

Supplementary Data 1

Supplementary Data 2

Reporting Summary

## Data Availability

Source data underlying the graphs in the main figures are available in Supplementary Data 1.
