# Supplementary material for: MYPT1 reduction is a pathogenic factor of erectile dysfunction
Source: Commun Biol. 2022 Jul 25;5:744. doi: 10.1038/s42003-022-03716-y (PMC9314386; doi:10.1038/s42003-022-03716-y)
Supplement: Supplementary file 1 — Supplementary Information [file 42003_2022_3716_MOESM1_ESM.pdf]

**Supplementary Material**

**MYPT1 reduction is a pathogenic factor of erectile dysfunction**

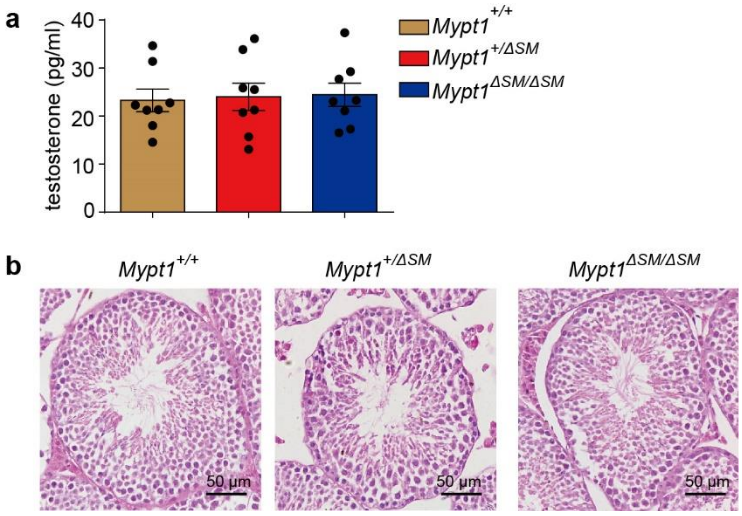


**Supplementary Figure 1. The phenotype of testis from different genotypic mice.** Equal level of testosterone (T) (**a**) (n=8) and H-E staining of testis (**b**) from different genotypic mice. The bars indicate the mean values ± SEM; p>0.05; *One-way ANOVA*.

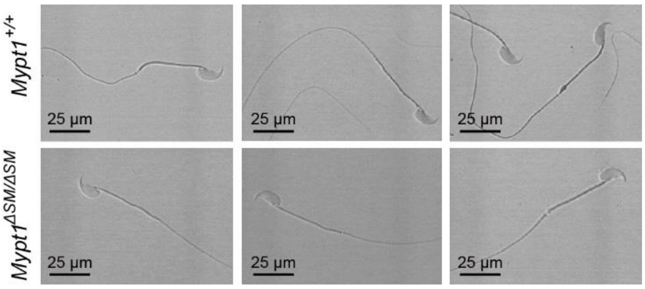


**Supplementary Figure 2. The morphological of sperm from epididymis between *Mypt1*$^{+/+}$ and *Mypt1*$^{\Delta SM/\Delta SM}$ mice.**

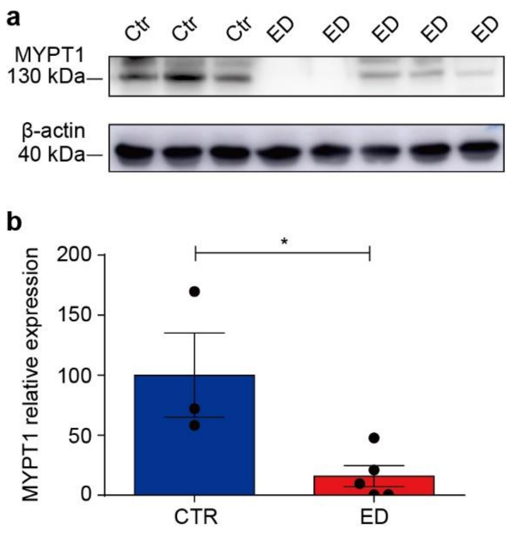


**Supplementary Figure 3. Decreased MYPT1 protein expression was detected in penile smooth muscle from ED patients. a**, CC biopsies from five patients with ED and three patients with penile cancer were collected and sampled for a Western blot assay. β-actin was used as the internal loading control. Panel A shows the MYPT1 expression in CC. **b**, Quantification of relative MYPT1 level with respect to β-actin (Control, n=3; ED, n=5); *$p<0.05$; *t-test*.

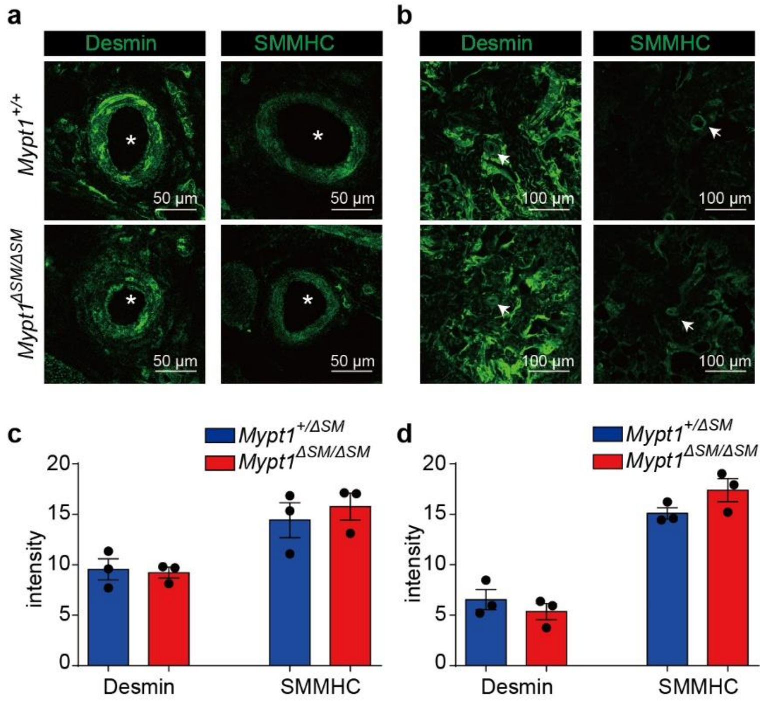


**Supplementary Figure 4. The fibrous fibers were detected in penis. a-b,** Immunofluorescence staining showed the expression of desmin and SMHHC of dorsal artery (**a,** asterisk) and CC (**b,** arrow) from *Mypt1*$^{+/+}$ and *Mypt1*$^{\Delta SM/\Delta SM}$ mice. **c-d,** Quantification of immunofluorescence intensity of desmin (c) and SMHHC (d) (n=3). The bars indicate the mean values ± SEM; p>0.05; *t*-test.

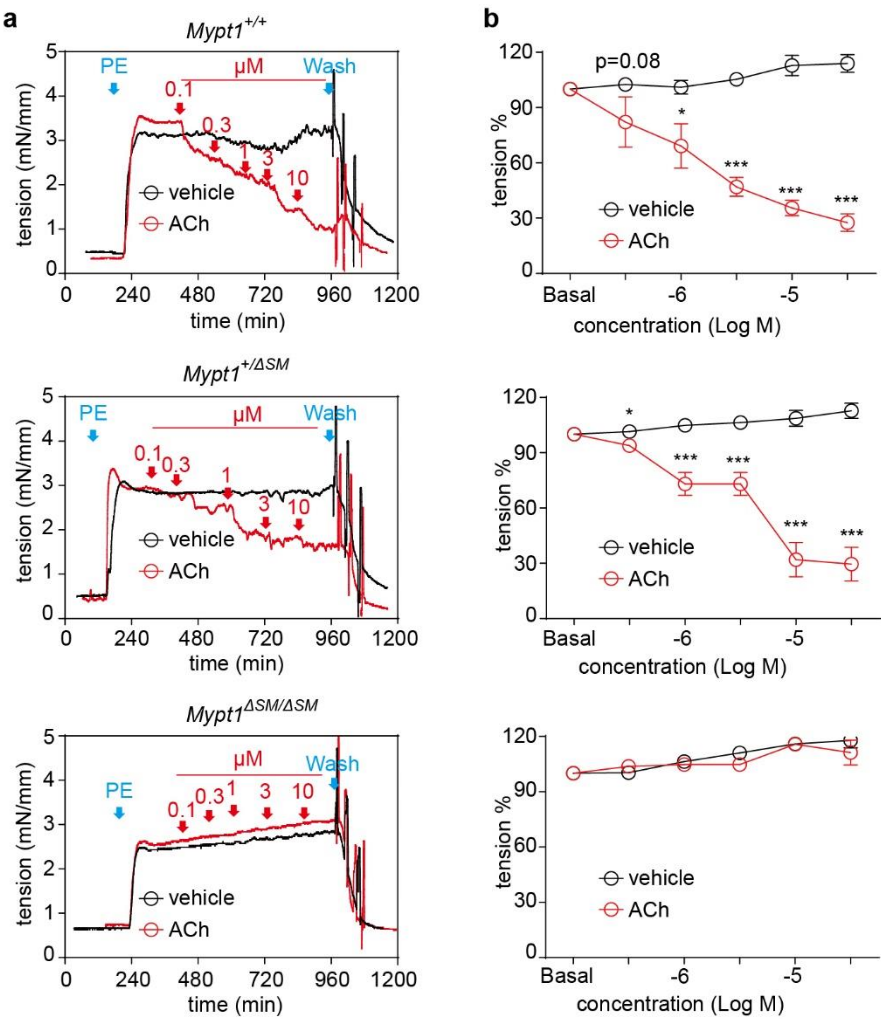


**Supplementary Figure 5. Relaxant effects of ACh on PE-evoked contraction of the dorsal artery. a,** Representative tracings of dorsal arteries precontracted using 10 μM PE and then exposed to ACh (0.1 μM to 10 μM) from *Mypt1*$^{+/+}$, *Mypt1*$^{+/\Delta SM}$ and *Mypt1*$^{\Delta SM/\Delta SM}$ mice. **b**, Quantification of the relative ratios of force relaxation by the reagents. (*Mypt1*$^{+/+}$, n=6; *Mypt1*$^{+/\Delta SM}$, n=11; *Mypt1*$^{\Delta SM/\Delta SM}$, n=3). The bars indicate the mean values ± SEM; *p<0.05, ***p<0.001; *pair t*-test.

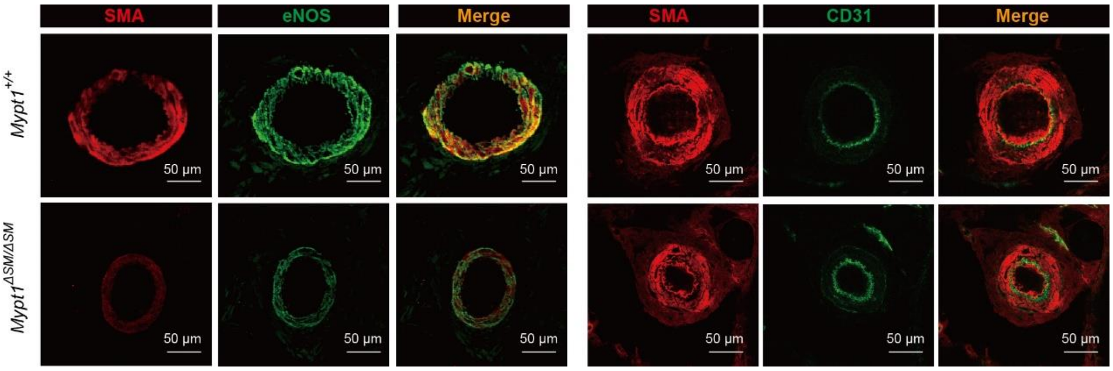


**Supplementary Figure 6. Immunofluorescence staining of eNOS and CD31 in penial arteries from *Mypt1*$^{+/+}$ and *Mypt1*$^{\Delta SM/\Delta SM}$ mice.**

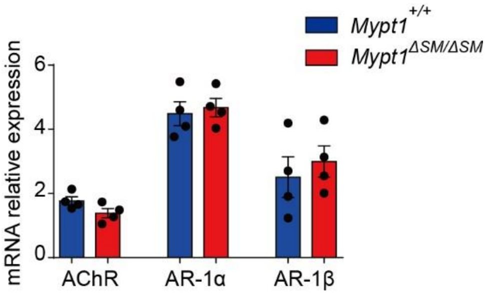


**Supplementary Figure 7. The mRNA expression of AChR, AR-1α and AR-1β.** The bars indicate the mean values ± SEM; n=4; $p>0.05$; *t*-test.

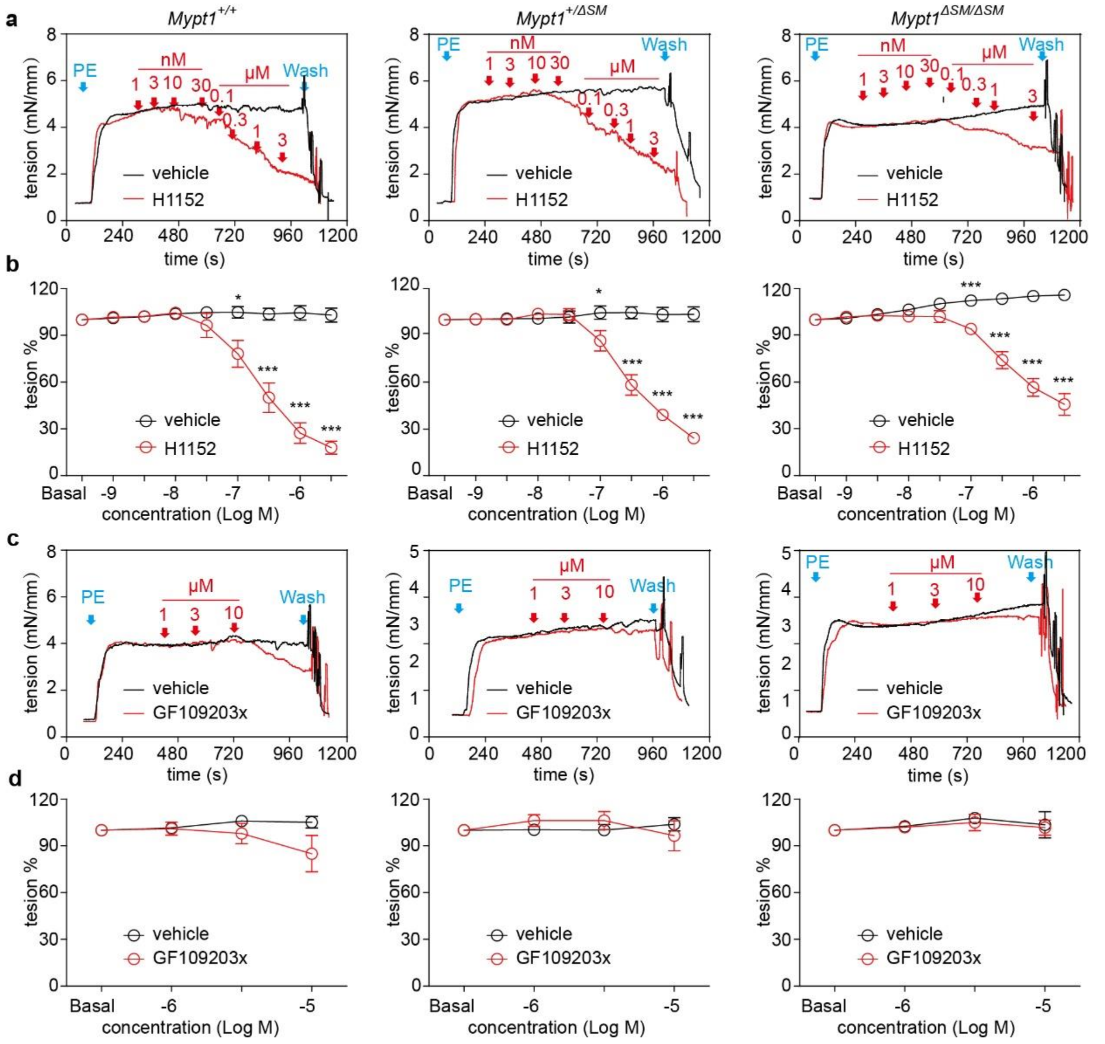


**Supplementary Figure 8. Relaxant effects of H1152 and GF109203x on PE-evoked contraction of the dorsal artery. a** and **c**, Representative tracings of dorsal arteries precontracted using 10 μM PE and then exposed to H1152 (1 nM to 3 μM) (**a**) or GF109203X (10 μM to 3 μM) (**c**). **b** and **d**, Quantification of the relative ratios of force relaxation by the reagents (n=6). The bars indicate the mean values ± SEM; *p<0.05, ***p<0.001; *pair t*-test.

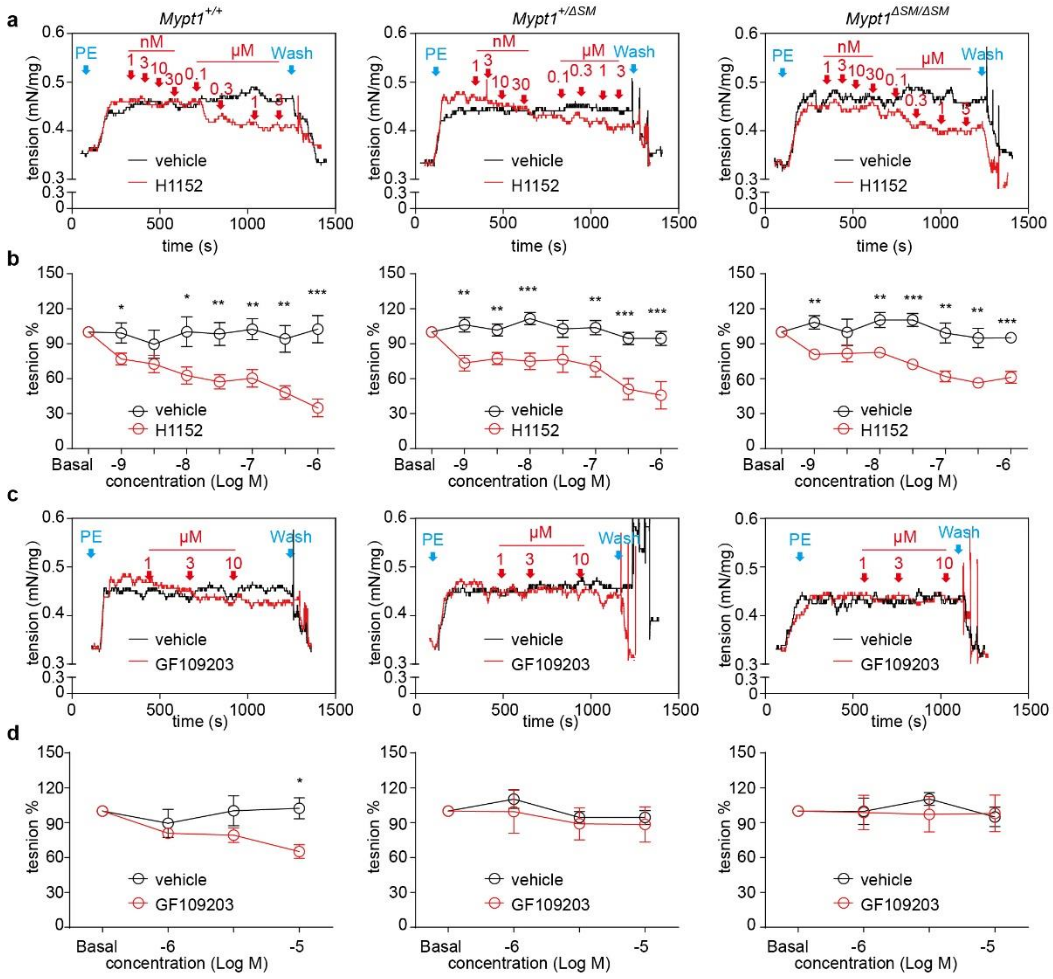


**Supplementary Figure 9. Relaxant effects of H1152 and GF109203x on PE-evoked contraction of the CC. a** and **c**, Representative tracings of CC precontracted using 10 µM PE and then exposed to H1152 (1 nM to 3 µM) (**a**) and GF109203X (10 µM to 3 µM) (**c**). **b** and **d**, Quantification of the relative ratios of force relaxation by the reagents. (Panel b: *Mypt1*$^{+/+}$, n=10; *Mypt1*$^{+/\Delta SM}$, n=10; *Mypt1*$^{\Delta SM/\Delta SM}$, n=6; Panel d: *Mypt1*$^{+/+}$, n=10; *Mypt1*$^{+/\Delta SM}$, n=10; *Mypt1*$^{\Delta SM/\Delta SM}$, n=6) The bars indicate the mean values ± SEM; *p<0.05, **p<0.01, ***p<0.001; *pair t*-test.

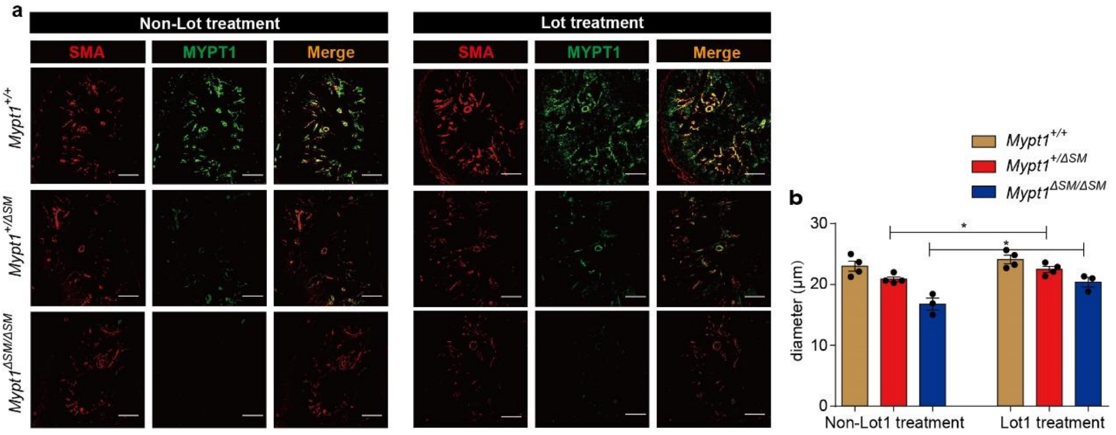


**Supplementary Figure 10. Lotusine treatment led to elevated MYPT1 expression and an enlarged diameter of the central artery of the CC. a**, Immunofluorescence staining showed an increase in MYPT1 expression and the diameter of the penile central artery after lotusine treatment. **b**, Quantitation of the central artery diameters. SMA (red); MYPT1 (green) (non-Lot1: *Mypt1*$^{+/+}$, n=4; *Mypt1*$^{+/\Delta SM}$, n=4; *Mypt1*$^{\Delta SM/\Delta SM}$, n=3; Lot1: *Mypt1*$^{+/+}$, n=4; *Mypt1*$^{+/\Delta SM}$, n=4; *Mypt1*$^{\Delta SM/\Delta SM}$, n=3). The bars indicate the mean values ± SEM; *p<0.05 by *t-test*; “arrow” represents CC.

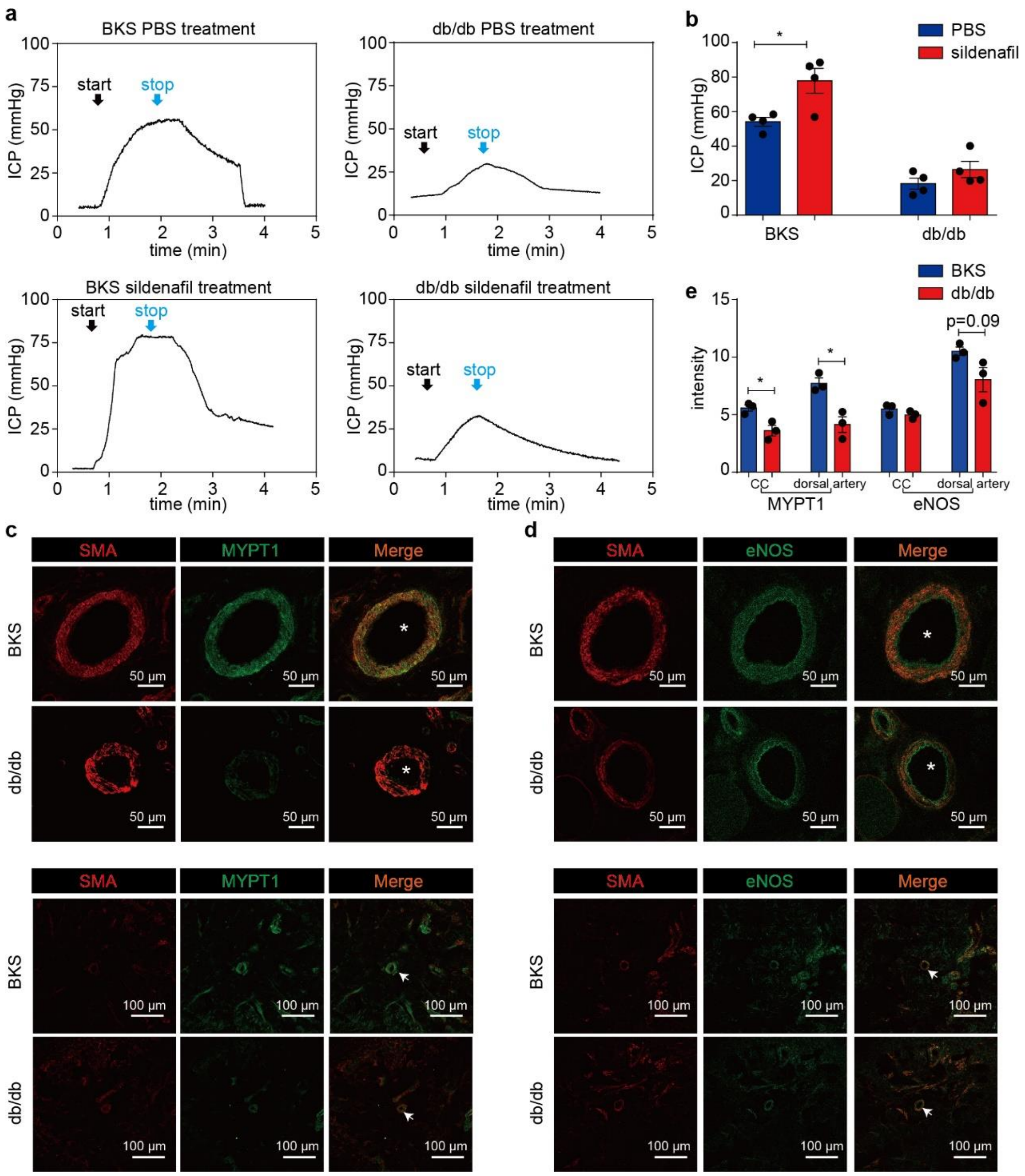


**Supplementary Figure 11. The erectile function of db/db mice. a**, Representative ICP tracings after stimulation of penises with 5 V/12 Hz for 1 min from BKS and db/db mice (treated with or without sildenafil) (sildenafil: 2 mg/kg, *i.v.*). **b**, Quantification of the ICP values of 4 groups (n=4). **c-d**, Immunological staining of the dorsal artery (asterisk) and CC (arrow) in *db/db* showed reduced expression of eNOS (**c**) and comparable expression of eNOS (**d**) in both the dorsal artery and CC. **e,** Quantification of immunofluorescence intensity of MYPT1 (**c**) and eNOS (**d**) (n=3). The bars indicate the mean values ± SEM; *p<0.05; *t-test*.

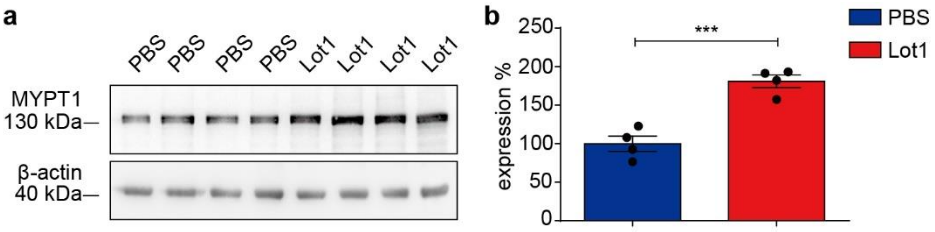


**Supplementary Figure 12. Expression of MYPT1 in aorta from db/db mice with or without Lot1 treatment.** db/db mice were injected *in vivo* with lotusine (5 mg/kg), and MYPT1 protein expression in the penises was measured by Western blotting. Protein data were analyzed with β-actin as the internal control (n=4). The bars indicate the mean values ± SEM; ***p<0.001; *t-test*.
