# Supplementary material for: MYPT1 reduction is a pathogenic factor of erectile dysfunction
Source: Commun Biol. 2022 Jul 25;5:744. doi: 10.1038/s42003-022-03716-y (PMC9314386; doi:10.1038/s42003-022-03716-y)
Supplement: Supplementary file 2 — Description of Additional Supplementary Files [file 42003_2022_3716_MOESM2_ESM.pdf]

Description of Additional Supplementary Files

File name: Supplementary Data 1
Description: The original data underlying the graphs in the main figures were in the Supplementary Data 1.

File name: Supplementary Data 2
Description: Unedited blot/gel images in main figures and supplementary figures were in the Supplementary Data 2.
