# Supplementary figures and images for: MYPT1 reduction is a pathogenic factor of erectile dysfunction
Source: Commun Biol. 2022 Jul 25;5:744. doi: 10.1038/s42003-022-03716-y (PMC9314386; doi:10.1038/s42003-022-03716-y)

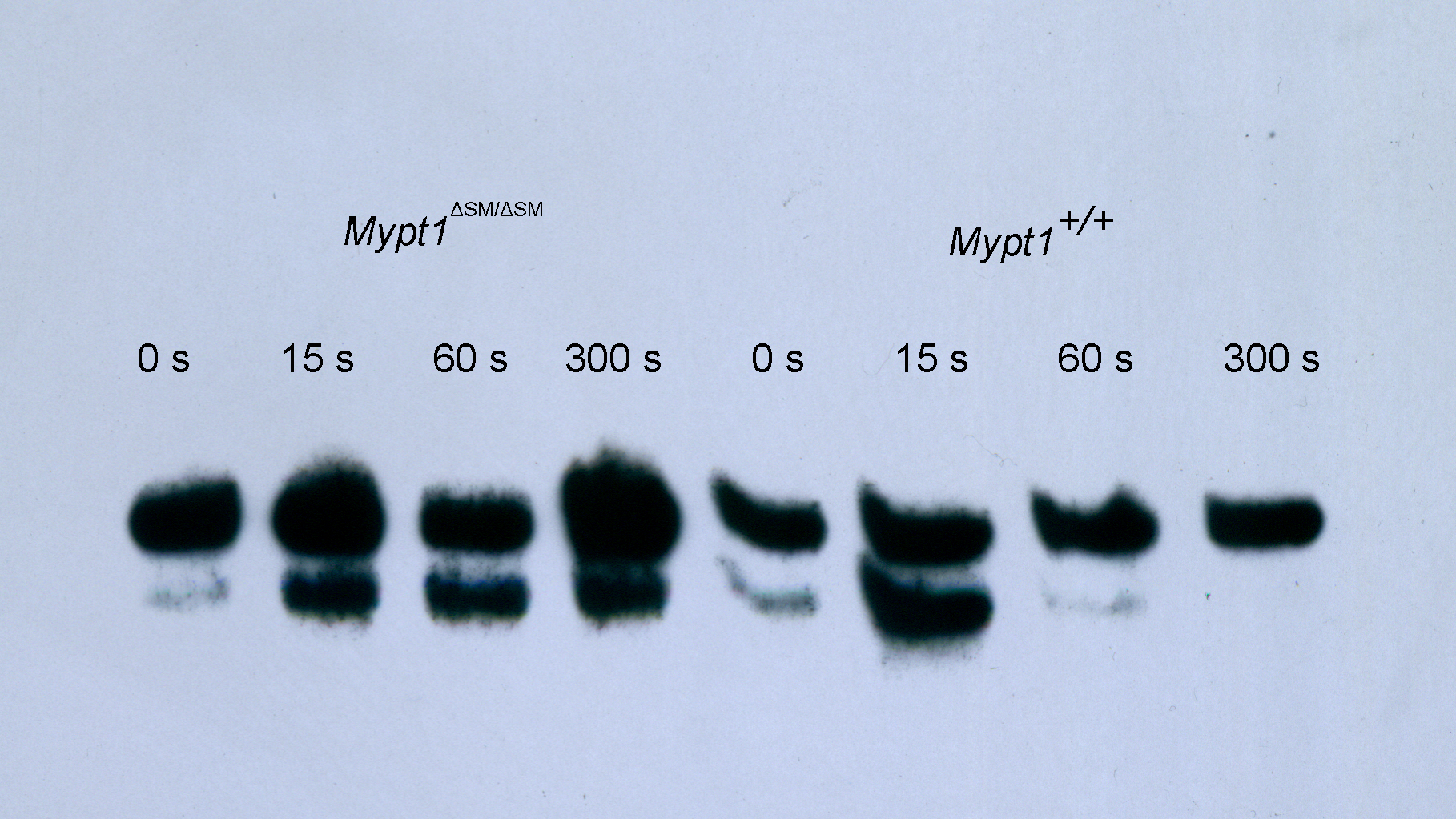

Supplement: Supplementary file 4 — Supplementary Data 2 [file 42003_2022_3716_MOESM4_ESM.zip › gel/Figure 3.jpg]

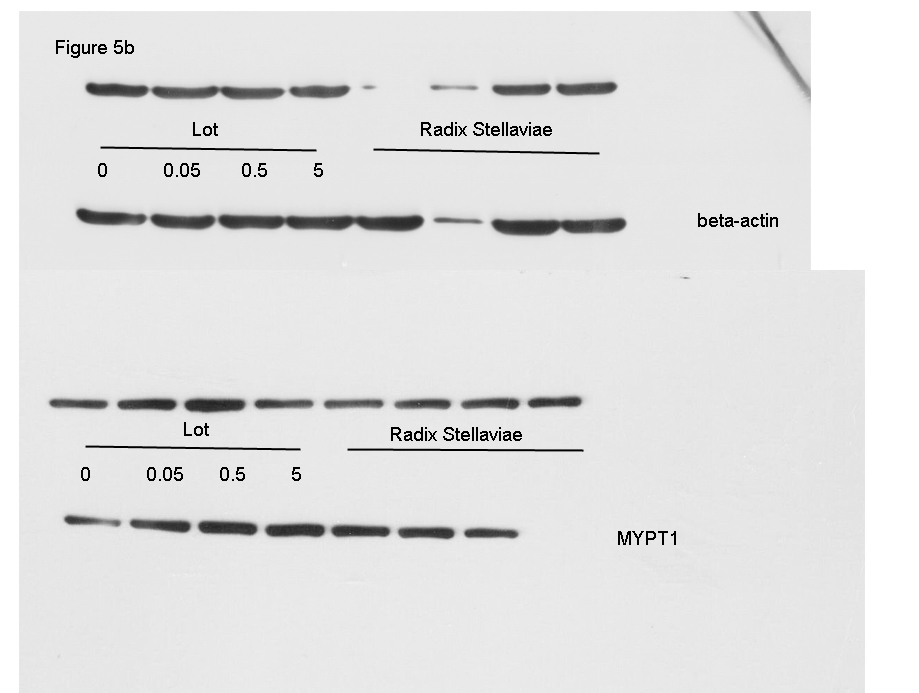

Supplement: Supplementary file 4 — Supplementary Data 2 [file 42003_2022_3716_MOESM4_ESM.zip › gel/Figure 5b.jpg]

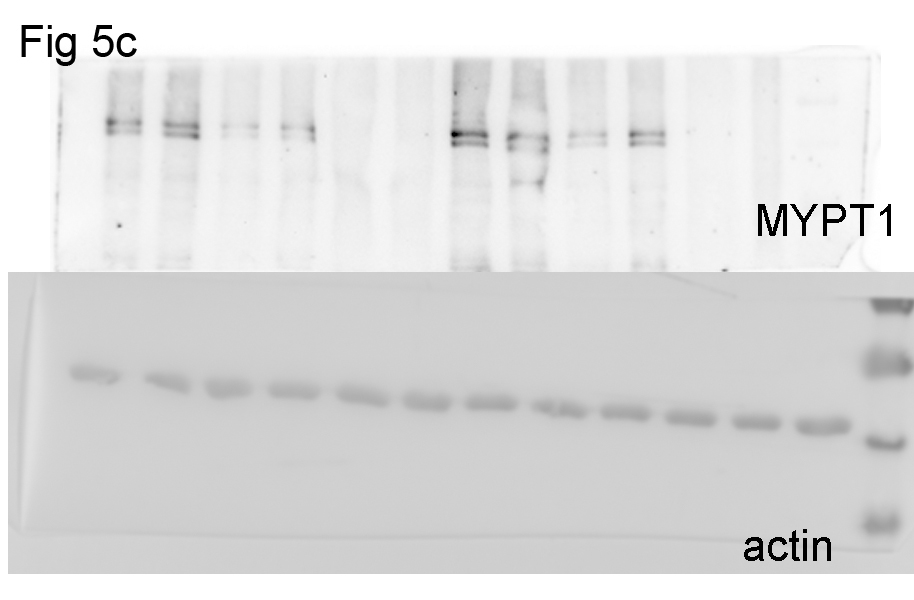

Supplement: Supplementary file 4 — Supplementary Data 2 [file 42003_2022_3716_MOESM4_ESM.zip › gel/Figure 5c.jpg]

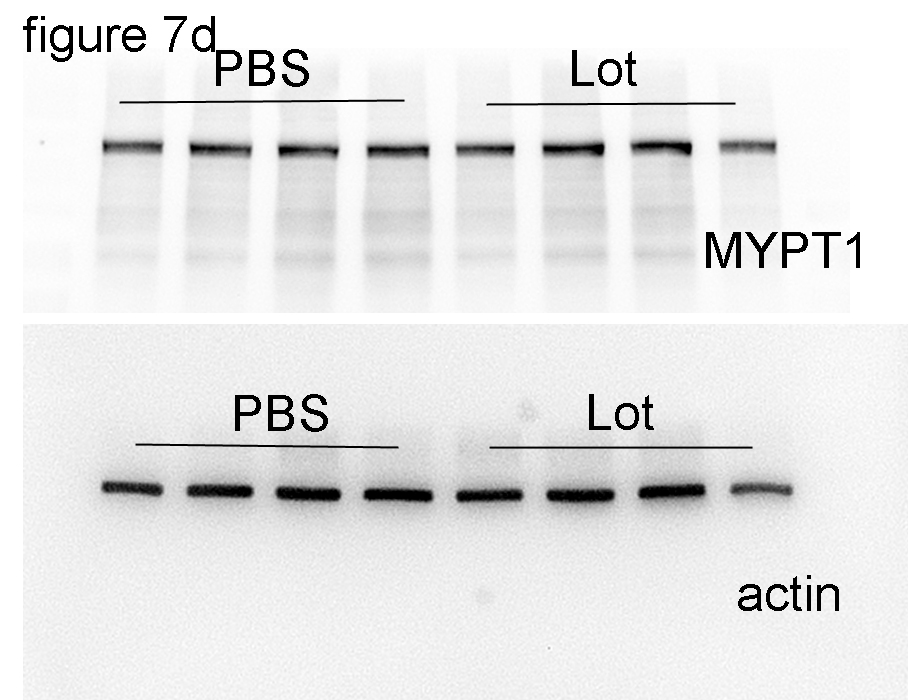

Supplement: Supplementary file 4 — Supplementary Data 2 [file 42003_2022_3716_MOESM4_ESM.zip › gel/Figure 7d.jpg]

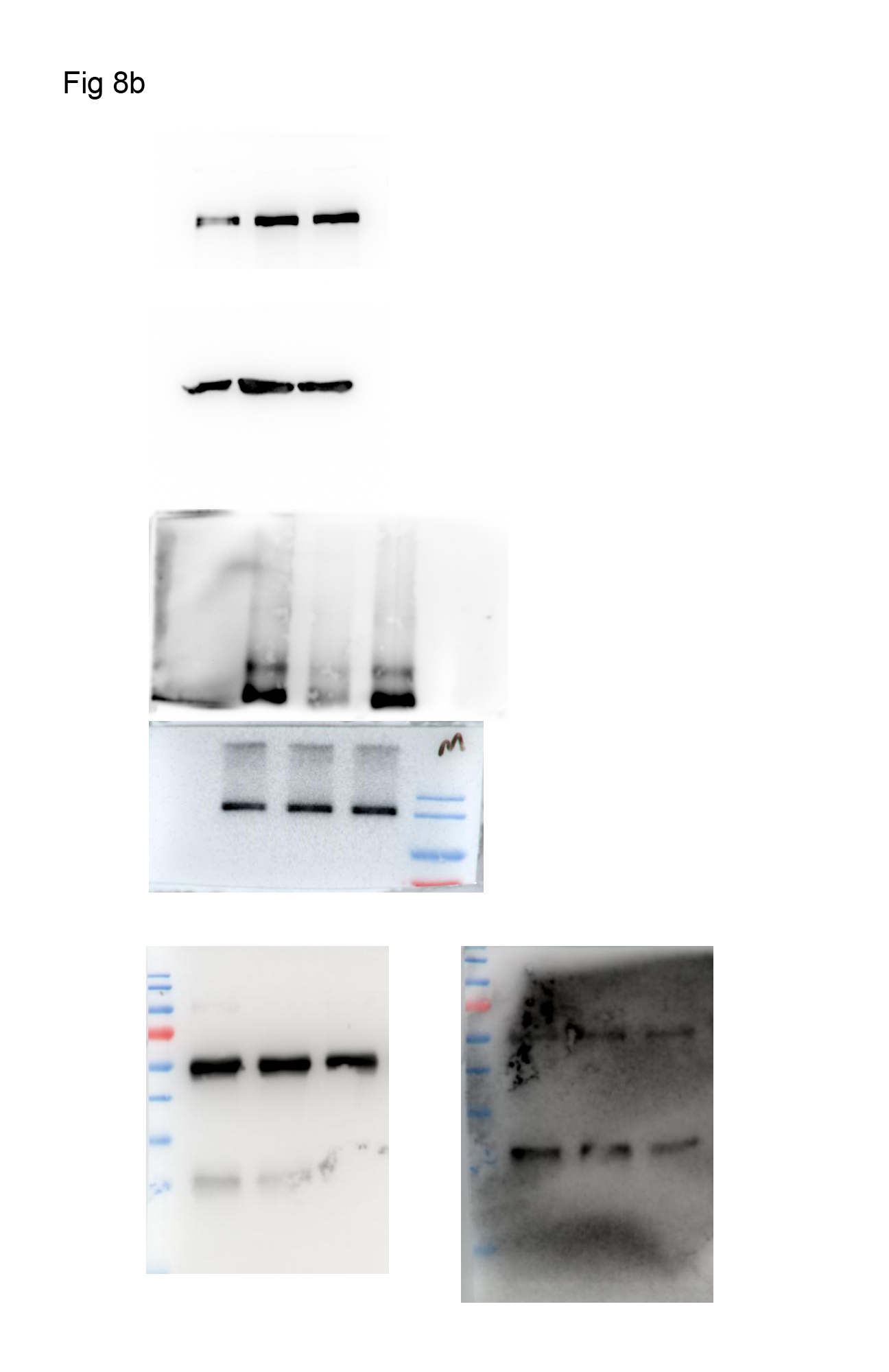

Supplement: Supplementary file 4 — Supplementary Data 2 [file 42003_2022_3716_MOESM4_ESM.zip › gel/Figure 8b.jpg]

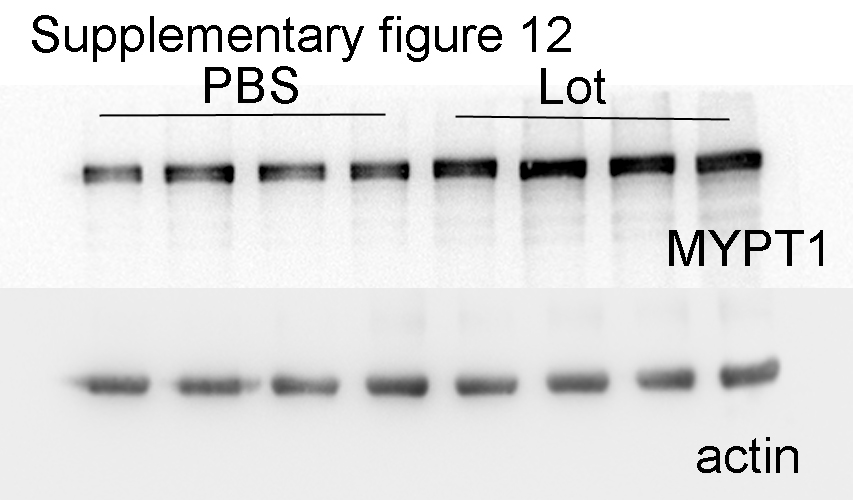

Supplement: Supplementary file 4 — Supplementary Data 2 [file 42003_2022_3716_MOESM4_ESM.zip › gel/Supplementary figure 12.jpg]

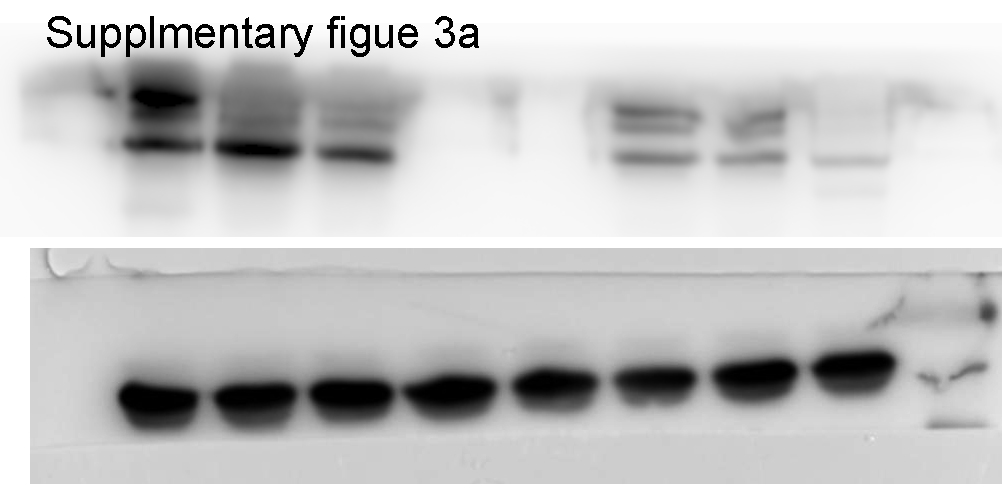

Supplement: Supplementary file 4 — Supplementary Data 2 [file 42003_2022_3716_MOESM4_ESM.zip › gel/Supplementary figure 3.jpg]
