# Supplementary material for: MYPT1 reduction is a pathogenic factor of erectile dysfunction
Source: Commun Biol. 2022 Jul 25;5:744. doi: 10.1038/s42003-022-03716-y (PMC9314386; doi:10.1038/s42003-022-03716-y)
Supplement: Supplementary file 5 — Reporting Summary [file 42003_2022_3716_MOESM5_ESM.pdf]

nature portfolio

Corresponding author(s): COMMSBIO-20-3318D

Last updated by author(s): Jun 29, 2022

Reporting Summary

Nature Portfolio wishes to improve the reproducibility of the work that we publish. This form provides structure for consistency and transparency in reporting. For further information on Nature Portfolio policies, see our Editorial Policies and the Editorial Policy Checklist.

Statistics

For all statistical analyses, confirm that the following items are present in the figure legend, table legend, main text, or Methods section.

n/a	Confirmed	
☐	☒	The exact sample size (*n*) for each experimental group/condition, given as a discrete number and unit of measurement
☐	☒	A statement on whether measurements were taken from distinct samples or whether the same sample was measured repeatedly
☐	☒	The statistical test(s) used AND whether they are one- or two-sided<br>*Only common tests should be described solely by name; describe more complex techniques in the Methods section.*
☒	☐	A description of all covariates tested
☒	☐	A description of any assumptions or corrections, such as tests of normality and adjustment for multiple comparisons
☐	☒	A full description of the statistical parameters including central tendency (e.g. means) or other basic estimates (e.g. regression coefficient) AND variation (e.g. standard deviation) or associated estimates of uncertainty (e.g. confidence intervals)
☒	☐	For null hypothesis testing, the test statistic (e.g. *F*, *t*, *r*) with confidence intervals, effect sizes, degrees of freedom and *P* value noted<br>*Give P values as exact values whenever suitable.*
☒	☐	For Bayesian analysis, information on the choice of priors and Markov chain Monte Carlo settings
☒	☐	For hierarchical and complex designs, identification of the appropriate level for tests and full reporting of outcomes
☒	☐	Estimates of effect sizes (e.g. Cohen's *d*, Pearson's *r*), indicating how they were calculated

Our web collection on statistics for biologists contains articles on many of the points above.

Software and code

Policy information about availability of computer code

Data collection	Chart 5.0 software; ADInstruments, Colorado Springs<br>ALC Non-invasive Blood Pressure System, Shanghai Alcott Biotech<br>Confocal microscope, Olympus<br>Microscopic Imaging, DotSlide, Olympus<br>Biacore T200 instrument, GE Healthcare
Data analysis	SPSS 20.0 software<br>ImageJ2/FIJI

For manuscripts utilizing custom algorithms or software that are central to the research but not yet described in published literature, software must be made available to editors and reviewers. We strongly encourage code deposition in a community repository (e.g. GitHub). See the Nature Portfolio guidelines for submitting code & software for further information.

Data

Policy information about availability of data

All manuscripts must include a data availability statement. This statement should provide the following information, where applicable:

- Accession codes, unique identifiers, or web links for publicly available datasets
- A description of any restrictions on data availability
- For clinical datasets or third party data, please ensure that the statement adheres to our policy

All data that support the finding during this study are included in this published article (and the supplementary information files), and these are available from the corresponding authors upon reasonable request.

Human research participants

Policy information about studies involving human research participants and Sex and Gender in Research.

Reporting on sex and gender	In the manuscript, we used the CC tissues undergoing the surgery. Therefore all the tissues were from the male.
Population characteristics	We have described the detailed information in the "Method Section".
Recruitment	The ED patients were undergoing penile prosthesis implantation surgery, and the control patients were undergoing penile carcinoma surgery. The patients' age were from 22 to 62-year-old. All the patients were diagnosed by IIEF and AVSS. These information were described in the "Method" Section.
Ethics oversight	All the experiments were approved by the Research Ethics Committee of Beijing Hospital and Research Ethics Committee of Nanjing Jinling Hospital (2020DZGZRZX-094), and all patients provided written informed consent.

Note that full information on the approval of the study protocol must also be provided in the manuscript.

Field-specific reporting

Please select the one below that is the best fit for your research. If you are not sure, read the appropriate sections before making your selection.

☒ Life sciences ☐ Behavioural & social sciences ☐ Ecological, evolutionary & environmental sciences

For a reference copy of the document with all sections, see nature.com/documents/nr-reporting-summary-flat.pdf

Life sciences study design

All studies must disclose on these points even when the disclosure is negative.

Sample size	No pre-specified effect size could be determined in advance. 3-17 mice were used to obtain a statistically significant difference.
Data exclusions	No data were excluded from the study.
Replication	The experimental findings were reliably reproducible as is shown in the paper and described above.
Randomization	This is not relevant to our study, because we used knockout mice line and divided them into different groups according their genotypic results in the same litter.
Blinding	We didn't know the genotypic results while doing our experiments. We did not match the experimental and genotypic results until the statistical analysis.

Reporting for specific materials, systems and methods

We require information from authors about some types of materials, experimental systems and methods used in many studies. Here, indicate whether each material, system or method listed is relevant to your study. If you are not sure if a list item applies to your research, read the appropriate section before selecting a response.

Materials & experimental systems

n/a	Involved in the study	
☐	☒	Antibodies
☐	☒	Eukaryotic cell lines
☒	☐	Palaeontology and archaeology
☐	☒	Animals and other organisms
☒	☐	Clinical data
☒	☐	Dual use research of concern

Methods

n/a	Involved in the study	
☒	☐	ChIP-seq
☒	☐	Flow cytometry
☒	☐	MRI-based neuroimaging

Antibodies

Antibodies used	anti-MYPT1: Cat. No. 22117-1-AP, Lot: 00055572, Proteintech<br>anti-smooth muscle β-actin: Cat. No.A5441, Sigma<br>anti-eNOS: Cat. BS3571, Lot: XCJ36131, Bioworld<br>anti-smooth muscle α-actin: Cat.No.ab7817, Lot: gr3356520, Abcam<br>anti-desmin: Cat. No. 21404-1-AP, Proteintech<br>anti-SMHHC: Cat. No. 16520-1-AP, Proteintech<br>anti-CD31: Cat.No.550274, Lot: 7131993, BD Biosciences<br>Goat anti-Rabbit IgG (H+L) Secondary Antibody, HRP conjugate: Cat.No.31460, Thermo<br>Alexa Fluor 488-conjugated donkey anti-mouse: Cat.No.A-21202, Thermo<br>Alexa Fluor 568-conjugated goat anti-mouse: Cat.No.A21043, Invitrogen
Validation	anti-RLC was used in our previous work: He, W.Q. et al. Gastroenterology. 2013; Qiao, Y.N. et al. JBC. 2014; Zhao, W. et al. CMGH. 2019.<br>The information of anti-RLC antibody is in the reference: Isotani, E. et.al. PNAS. 2004

Eukaryotic cell lines

Policy information about cell lines and Sex and Gender in Research

Cell line source(s)	A7R5 cell line
Authentication	A7R5 cell line was used in our previous work: Wei, L.S. et al. JBC. 2020
Mycoplasma contamination	The cell lines were not tested for mycoplasma contamination.
Commonly misidentified lines<br>(See ICLAC register)	None.

Animals and other research organisms

Policy information about studies involving animals; ARRIVE guidelines recommended for reporting animal research, and Sex and Gender in Research

Laboratory animals	C57BL/6, 8-15 weeks old (male or female), MYPT1 knockout mice, BKS and db/db mice were contracted in Model Animal Research Center of Nanjing University
Wild animals	No wild animal were used in this study.
Reporting on sex	In this research, all mice were male mice. The male mice were used to detect the function of penile arteries and erection.
Field-collected samples	No field-collected samples were used in this study.
Ethics oversight	All animal protocols were approved by the Animal Care and Use Committee of Model Animal Research Center of Nanjing University (institutional permission #ZMS-24). The sample collection was approved by the Research Ethics Committee of Beijing Hospital and Research Ethics Committee of Nanjing Jinling Hospital (2020DZGZRZX-094).

Note that full information on the approval of the study protocol must also be provided in the manuscript.
